# *Mazus zhewanensis* (Mazaceae): A new species from Eastern China

**DOI:** 10.1371/journal.pone.0356991

**Published:** 2026-10-07

**Authors:** Ke-Lang Lou, Wen-Yuan Xie, Pan Xu, Hong-Wei Zhang, Feng Chen, Jun-Ping Li, Jin-Bao Pu, Zheng-Hai Chen, Wei-Qing Liang

**Affiliations:** 1 Zhejiang Academy of Traditional Chinese Medicine, Hangzhou, Zhejiang, China; 2 Tongde Hospital of Zhejiang Province, Hangzhou, Zhejiang, China; 3 Zhejiang Forest Resources Monitoring Center, Hangzhou, Zhejiang, China; 4 Administration of Zhejiang Qingliangfeng National Natural Reserve, Hangzhou, Zhejiang, China; 5 Yongkang Forestry Bureau, Yongkang, China; 6 Zhejiang Forestry Survey Planning and Design Co., Ltd, Hangzhou, Zhejiang, China; 7 State Key Laboratory for Quality Assurance and Sustainable Use of Dao-di Herbs, Beijing, China; Zhejiang Agriculture and Forestry University: Zhejiang A and F University, CHINA

## Abstract

A new species, *M. zhewanensis* **sp. nov.** (Mazaceae), is described from East China. Morphologically, it is similar to *M. spicatus* but differs in having elliptic cauline leaves with shallowly and densely serrate margins (vs. obovate to obovate-spatulate with incised-serrate margins) and a larger corolla. Phylogenetically, it is recovered as sister to *M. caducifer* with high support (**ML = 95%, BI = 1**). A key to the pubescent-fruited species of *Mazus* in China, including the new species, is also provided.

## Introduction

The genus *Mazus* Lour. (Mazaceae) comprises approximately 45 species, distributed primarily across temperate and subtropical Asia, as well as parts of Australia and New Zealand [1,2]. As currently circumscribed, the genus is characterized by small herbaceous plants with a distinctly two-lipped corolla, a palate with two longitudinal plaits, fibrous roots, and a capsule enclosed within the persistent calyx [1,3].

China is the center of diversity for *Mazus*, with more than 30 species [1]. Historically, the genus was placed in Scrophulariaceae, but molecular phylogenetic studies have since transferred it to the recently established family Mazaceae, in which it forms a clade with *Lancea* and *Dodartia* [4–6]. Within *Mazus*, early classifications recognized two sections based on stolon presence [7,8], this scheme was later expanded to three sections [1,9]. The monotypic section *Lanceifoliae* was recently segregated as the new genus *Puchiumazus* [10], while a revised infrageneric classification based on molecular and biogeographic evidence proposed two subgenera—*M.* subg. *Mazus* (Asia) and *M.* subg. *Notomazus* (Australia) [5]. A comprehensive taxonomic revision of the family worldwide was later provided by Lü [11].

Recent years have witnessed accelerated taxonomic discovery within the genus. Between 2016 and 2026, more than ten new species have been described from China [5,10,12–17], revealing substantial hidden diversity, particularly in eastern China. These include *M. sunhangii* from Hubei [12], *M. danxiacola* from Jiangxi [13], *M. unguiculatus* from Anhui [14], *M. motuoensis* from Xizang [15], *M. jingtaiensis* from Zhejiang and Fujian [16], and *M. jiangshiense* from Fujian [17]. Molecular phylogenetic analyses have resolved five major subclades within Asian *Mazus*, with a well-supported clade (subclade IV) containing *M. caducifer*, *M. spicatus*, and *M. xiuningensis*—highlighting the close affinities and complex evolutionary history among these taxa [5].

Eastern China has emerged as an important center of endemism for *Mazus*. In addition to the widespread taxa *M. stachydifolius*, *M. caducifer*, *M. pumilus*, *M. miquelii*, and *M. gracilis* [1,18–22], the region hosts a growing number of endemic species, including *M. fukienensis* [23], *M. wanmuliensis* [24], *M. jiangshiense* [17], *M. xiuningensis* [25], *M. apicaliracemosus* [26], *M. jingtaiensis* [16], and *M. unguiculatus* [14]. Species such as *M. saltuarius* [27] and *M. jingtaiensis* [16] have distributions spanning adjacent provinces, further underscoring the region‘s significance as a diversification center.

Despite these advances, species delimitation within *Mazus* remains challenging due to considerable morphological variability and overlapping diagnostic characters [7,8]. The genus exhibits substantial plasticity in vegetative and floral traits, which has historically led to taxonomic confusion and differing species concepts [1,5,7]. Recent integrative studies have emphasized the importance of combining morphological, molecular phylogenetic, and ecological evidence for robust species delimitation [5,13,14,17].

During recent field investigations in Zhejiang and Anhui provinces, we discovered an unknown *Mazus* taxon referable to section *Trichogynus*, characterized by a pubescent ovary and distinctively elongated stolons. Morphological examination, comparison with type material, and phylogenetic analyses based on chloroplast DNA regions (*rps16*, *rbcL*, and *trnL-trnF*) support its recognition as a distinct species.

Here, we describe this taxon as *Mazus zhewanensis* **sp. nov.**, provide diagnostic characters distinguishing it from related species, and present molecular evidence supporting its species status. A key to the pubescent-fruited species of *Mazus* in China is also provided.

## Materials and methods

### Morphological examination

We examined herbarium collections from PE, ZM, ZJMR, and 17 other herbaria (full list in S1 Table), supplemented by fresh field collections. In total, over 120 specimens representing more than 20 species of *Mazus* were studied, including type material of *M. spicatus*, *M. xiuningensis*, *M. jiangshiense*, and *M. caducifer*. For the new species, we measured ≥20 living plants in the field using digital calipers (0.1 mm accuracy), recording both qualitative (shape, color, indumentum) and quantitative (length, width, ratios) characters. A stereomicroscope (Olympus SZX16) was used to examine fine morphological features such as indumentum and serration margins. Field identifications were later verified against voucher specimens. Data for other taxa came from the literature and from direct examination of herbarium specimens. Voucher information for all material examined is provided in S1 Table.

### Taxon sampling

Fresh leaves from three populations were collected for DNA extraction. The leaves were placed in non-woven fabric bags and dehydrated in sealed bags with color-indicator silica gel. Voucher specimens from each population were deposited in the Herbarium of Medicinal Resources, Zhejiang Academy of Traditional Chinese Medicine (ZJMR); the Zhejiang Museum of Natural History (ZM); and the Institute of Botany, Chinese Academy of Sciences (PE).

### DNA extraction, PCR amplification, and sequencing

To elucidate the phylogenetic relationships and taxonomic placement of the proposed new species within *Mazus*, we conducted phylogenetic analyses based on three chloroplast DNA regions (*rbcL*, *rps16*, and *trnL-trnF*) and the nuclear ribosomal internal transcribed spacer (ITS). Polymerase chain reaction (PCR) amplification was performed using the following primers: *rbcL*, RH1/Z1352RC [28]; *rps*16, *rps*16-2F/*rps*16-R3 [29]; *trnL-trnF*, the c/f primers [30]; and ITS, ITS1/ITS4 [31]. Laboratory protocols and downstream data processing followed a published workflow [17]. To further resolve the phylogenetic placement of *M. zhewanensis* within the *M. caducifer* complex, we expanded our sampling of *M. caducifer* by collecting and sequencing additional specimens from three populations in Zhejiang Province. These newly obtained sequences were combined with publicly available data from GenBank to generate an expanded dataset for phylogenetic reconstruction.

### Genetic distance and molecular diagnostic characters

Genetic distances between and within species were calculated using the ape package [32] in R [33], based on the Kimura 2-parameter (K2P) model [34]. To identify fixed nucleotide diagnostic sites between *M. zhewanensis* and its closest relative *M. caducifer*, we examined aligned chloroplast sequences (*rps16*, *rbcL*, and *trnL-trnF*) using the seqinr package in R [33,35].

### Phylogenetic analyses

To determine the phylogenetic position of the newly discovered species, sequence data for *rbcL*, *rps*16, *trnL-trnF* and *ITS* were obtained from GenBank and supplemented with sequences generated in this study. *Dodartia orientalis* L [36]., *Lancea tibetica* Hook.f. & Thomson [37], and *Puchiumazus lanceifolius* (Hemsl.) Bo Li, D.G. Zhang & C.L. Xiang [10] were chosen as outgroups following Li et al. [13] and Chen et al. [17]. Accession numbers for all taxa are listed in Table 1.

**Table 1:** Taxon and GenBank accession numbers of the specimens.

Taxa	rbcL	rps16	trnL-trnF	ITS
*Dodartia orientalis* L.	JQ342984	JQ342982	JQ342981	JQ342980
*Lancea tibetica* Hook.f. & Thomson	MF786661	FJ172699	FJ172685	FJ172736
*M. alpinus* Masam.	KX783481	KX783501	KX783520	MK192641
*M. caducifer* Hance	KX783477	KX783497	MK266455	MK192664
*M. caducifer* Hance	KX783487	KX783506	KX783526	MK192659
*M. caducifer* Hance	PZ707393*	PZ717420*	PZ707396*	PZ683389*
*M. caducifer* Hance	PZ707394*	PZ717421*	PZ707397*	PZ683390*
*M. caducifer* Hance	PZ707395*	PZ717422*	PZ707398*	PZ683391*
*M. celsioides* Hand.-Mazz.	KX783486	MK266366	KX783525	MT703875
*M. danxiacola* Bo Li & B.Chen	ON323565	ON323567	ON323569	ON286711
*M. delavayi* Bonati	KX783482	KX783502	KX783521	/
*M. fauriei* Bonati	KX783479	KX783499	KX783518	MK192640
*M. fruticosus* Bo Li, D.G.Zhang & C.L.Xiang	/	KX783490	KX783509	MK192660
*M. fukienensis* P.C.Tsoong	MT712054	MT712059	MT712064	MT707248
*M. gracilis* Hemsl.	FJ172729	FJ172701	FJ172687	FJ172738
*M. henryi* P.C.Tsoong	MT712055	MT712060	MT712065	MT707246
M*. humilis* Hand.-Mazz.	PQ824921	MK266401	MK266458	MK192667
M*. jiangshiense* Y.B.Chen, Xin Y. Chen & Liang Ma	OP616018	OP616019	OP616020	OP605381
*M. longipes* Bonati	KX783474	KX783494	KX783513	MK192652
*M. miquelii* Makino	KX783475	KX783495	MK266423	MK192637
*M. motuoensis* W.B.Ju, Bo Xu bis & X.F.Gao	OQ383431	/	OQ383430	OP720888
*M. novaezeelandiae* W.R.Barker	KX783469	KX783489	KX783508	MK192676
*M. omeiensis* H.L.Li	KX807209	KX807203	KX807208	MK192636
*M. procumbens* Hemsl.	KX783478	KX783498	KX783517	MK192647
*M. pulchellus* Hemsl.	KX783472	KX783492	KX783511	MK192638
*M. pumilio* R.Br.	KX783468	KX783488	KX783507	MK192671
*M. pumilus* (Burm.f.) Steenis	FJ172728	FJ172700	FJ172686	FJ172737
*M. radicans* Cheeseman	/	MK266404	MK266461	MK192669
*M. reptans* N.E.	HQ384872	HQ385147	AF479004	AF478940
*M. saltuarius* Hand.-Mazz.	MT712057	MT712062	MT712068	MT707245
*M. spicatus* Vaniot	FJ172730	FJ172703	MK266471	FJ172740
*M. stachydifolius* (Turcz.) Maxim.	EU348860	AJ609167	MT712070	MT703903
*M. sunhangii* D.G.Zhang & T.Deng	KX783484	KX783504	KX783523	/
*M. surculosus* D.Don	KX783473	KX783493	KX783512	/
*M. xiuningensis* X.H.Guo & X.L.Liu	MT712058	MT712063	MT712071	MT707247
*M. zhewanensis* Z.H. Chen, W.Y Xie et J.B. Pu	PX578853*	PX578856*	PX578859*	PX607764*
*M. zhewanensis* Z.H. Chen, W.Y Xie et J.B. Pu	PX578854*	PX578857*	PX578860*	PX607765*
*M. zhewanensis* Z.H. Chen, W.Y Xie et J.B. Pu	PX578855*	PX578858*	PX578861*	PX607766*

* = self-acquired in this study; the others are from GenBank. / = represents data deficient.

All phylogenetic analyses were conducted using PhyloSuite [38]. We employed three inference methods: Neighbor-Joining (NJ), Maximum Likelihood (ML), and Bayesian Inference (BI). For ML analyses, we used IQ-TREE 3.0.1 [39] implemented in PhyloSuite, with the best-fit substitution models selected by ModelFinder [40] and node support assessed using the Ultrafast Bootstrap [41] method with 1,000 replicates. BI was conducted using MrBayes [42] within PhyloSuite, with Markov Chain Monte Carlo (MCMC) chains run until convergence was achieved. NJ analyses were performed using MEGA X [43] via PhyloSuite, based on Kimura 2-parameter (K2P) distances with 1,000 bootstrap replicates. Additionally, we conducted separate phylogenetic analyses for the ITS and chloroplast datasets to assess cytonuclear discordance. The congruent results from these analyses were used to infer the evolutionary relationships among the species.

To quantitatively assess the congruence between the ITS and chloroplast datasets, we performed a partition homogeneity test (ILD) [44] implemented in PAUP* 4.0a [45], with 1,000 replicates, using parsimony as the optimality criterion. Gaps were treated as missing data, and heuristic searches were conducted with random stepwise addition (10 replicates) and tree-bisection-reconnection (TBR) branch-swapping.

### Nomenclature

The electronic version of this article in Portable Document Format (PDF) in a work with an ISSN or ISBN will represent a published work according to the International Code of Nomenclature for algae, fungi, and plants, and hence the any new names contained in the electronic publication of a PLOS ONE article are effectively published under that Code from the electronic edition alone, so there is no longer any need to provide printed copies.

In addition, the new name contained in this work has been submitted to IPNI, from where it will be made available to the Global Names Index. The IPNI LSIDs can be resolved and the associated information viewed through any standard web browser by appending the LSID contained in this publication to the prefix http://ipni.org/. The online version of this work is archived and available from the following digital repositories: PubMed Central, LOCKSS.

## Results and discussion

### Morphological comparison

*M. zhewanensis* is morphologically distinct from all pubescent-fruited *Mazus* species in China, characterized by erect stems, long stolons, elliptic cauline leaves with shallowly and densely serrate margins, and a relatively large corolla. Among these, it is most similar to *M. spicatus*, but the two are readily distinguished by the shape and margin of the cauline leaves: elliptic with shallow, dense serrations in *M. zhewanensis* versus obovate to obovate-spatulate with incised-serrate margins in *M. spicatus*.

Phylogenetically, *M. zhewanensis* is sister to *M. caducifer* (Fig 1). They differ markedly in stolons and calyx morphology: *M. zhewanensis* produces long, trailing stolons that root adventitiously at the nodes, whereas stolons are completely absent in *M. caducifer*; the calyx is campanulate in *M. zhewanensis* but infundibular in *M. caducifer*. *M. zhewanensis* is also distinguished from stoloniferous species without a distinct erect stem, such as *M. xiuningensis* and *M. jiangshiense*, by its well-developed erect stem and corresponding leaf morphology. For a full comparison, see Table 2 and the revised key.

**Table 2:** Comparison between *M. zhewanensis* and similar taxa.

Character		*M. zhewanensis* ^$^	*M. caducifer* ^δ^	*M. spicatus* ^α^	*M. xiuningensis* ^β^	*M. jiangshiense* ^γ^
Basal Leaves		Often withering at anthesis.	Withering at anthesis.	Few and early withering at anthesis.	Persistent at anthesis.	Persistent at anthesis.
Stem	Morpholog	Stems erect; stolons ascending arising from base of erect stems and inflorescences.	Stems erect; stolons arising from the base of the plant.	Stems erect; stolons arising from the base of the plant.	Diffuse, with stolons only.	Diffuse, with stolons only.
	Indumentu	Densely white villous.	White villous.	With white or pale rusty tomentose.	Densely pubescent.	Densely white villous.
	Stolon Rooting Condition	At late flowering stage, the tips of ascending stems **root upon contact with the ground** and develop into rosette plants.	**Ascending stems do not root.**	Ascending stems produce adventitious roots when touching the ground.	Stolons produce adventitious roots upon contact with the ground.	Stolons root at nodes.
Inflorescence	Insertion	At apex of erect stems.	At apex of ascending stems.	At apex of erect stems and ascending stems.	At apex of procumbent stems.	On stoloniferous stem.
		11–28 cm long, with (6–)10–20 flowers; pedicel: 6–10 mm.	Up to 35 cm long, with 6–23 flowers.	6.5–20 cm long, with 7–10 flowers.	10-12 cm long,with 3–10 flowers.	To 17.0 cm or longer, multiflowered to 20, with 7–17 flowers;.
Calyx		**Campanulate**, 8–9 mm in fruit, sparsely white-strigose and glandular-pubescent externally; lobes lanceolate, nearly equal to or slightly longer than tube.	**Funnelform**, ca. 13 mm long and 1 cm wide in fruit, villous externally; calyx lobes ovate-lanceolate, nearly as long as the tube.	Campanulate, 5–8 mm in fruit, margin short-ciliate; lobes triangular-lanceolate, acute, nearly equal to tube.	Broadly campanulate, 3–8 mm in fruit, villous externally; lobes ovate, shorter than the tube.	Infundibular (funnelform), 3.0–4.0 mm, slightly accrescent in fruit, villous on both surfaces; lobes 5, lanceolate, acute, longer than tube.
Corolla		**1.4-2.0 cm** long; upper lip, lobes narrowly triangular, entire.	1.2–3.5 cm long; upper lip lobes ovate-lanceolate, entire.	**0.8–1.2 cm** long; upper lip lobes narrowly acute, entire.	1.3–1.7 cm long; upper lip lobe apices irregularly dentate.	0.9–1.1 cm long; upper lip bilobed, lobes lanceolate, acute, entire.
Filaments		Anterior pair linear.	Anterior pair linear.	Anterior pair linear.	Anterior pair linear.	Anterior pair curved.
Ovary		Densely short-pilose intermixed with sparse long-hirsute hairs at apex.	Upper half with hairs.	Hirsute throughout.	Densely clavate-glandular hairy throughout.	Villous at apex.
Phenology		Flowering: April–August; Fruiting: June–October.	Flowering: April–May; Fruiting: June–August.	Flowering: May–June; Fruiting: July–August.	Flowering: June–July; Fruiting: August–September.	Flowering: June–July; Fruiting: August–September.
Distribution		Northwestern Zhejiang (Lin’an), Southeastern Anhui (Jixi).	Anhui, Zhejiang, Jiangxi	Hunan, Hubei, Guangxi, Guizhou, Sichuan, and Southern Shaanxi.	Southeastern Anhui (Xiuning).	Northwest Fujian (Shaowu).

Data sources: $, based on measurements of ≥20 individuals from populations in Zhejiang and Anhui (vouchers deposited at ZJMR); α, Vaniot (1905); β, Guo & Liu (1990); γ, Chen et al. (2024); δ, Hance (1882), supplemented by own measurements.

**Fig 1:**
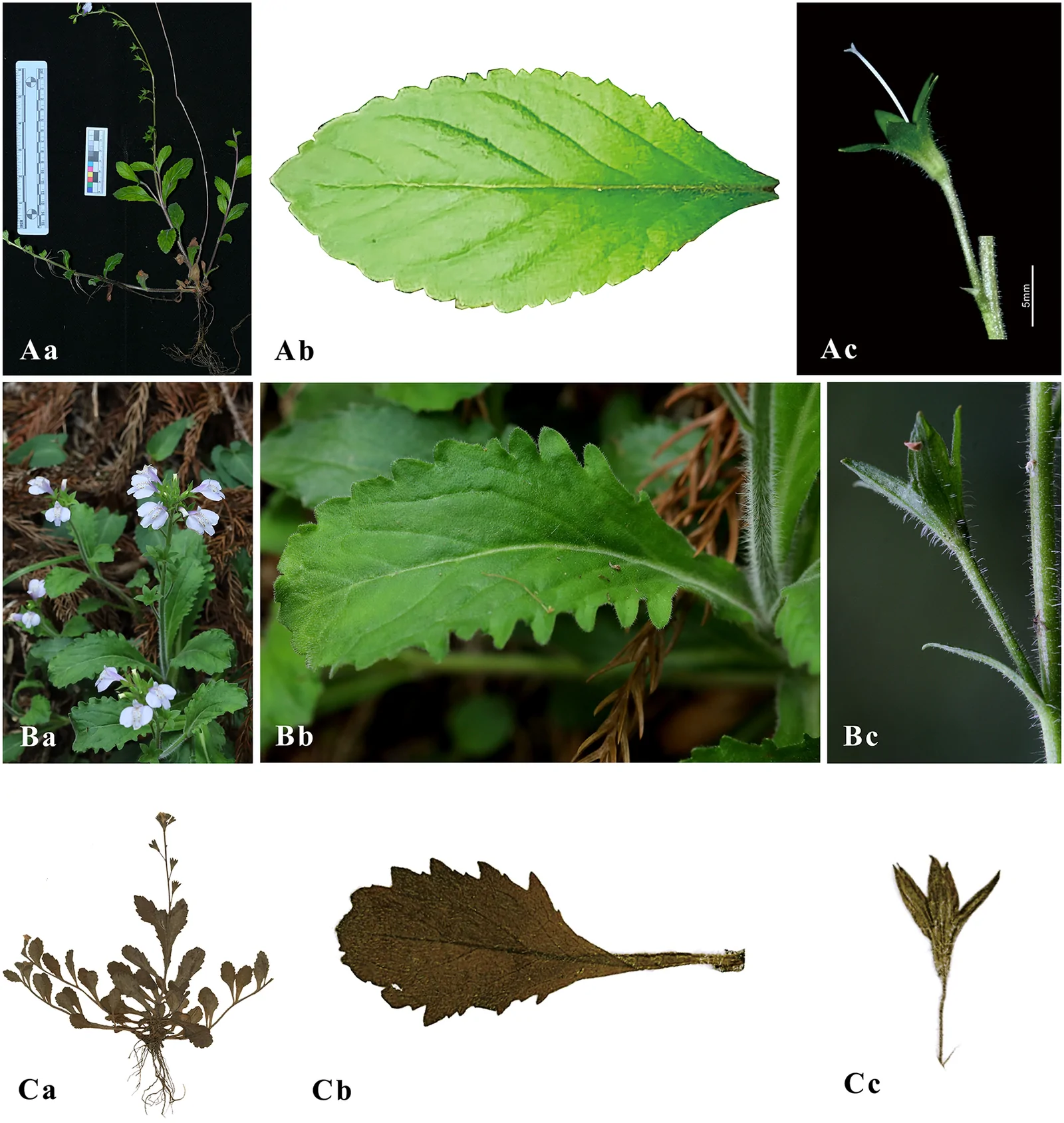
Morphological comparison of *M. zhewanensis* (A), *M. caducifer* (B), and *M. spicatus* (C). (Aa). habit; (Ab). leaf and serration; (Ac). calyx. (Ba). habit; (Bb). leaf and serration; (Bc). calyx. (Ca). habit (holotype of M. spicatus, E00284108, Bodinier #1593); (Cb). leaf and serration (syntype of M. bodinieri, E00284109, Cavalerie #809); (Cc). calyx (same specimen).

### Molecular phylogenetic relationship

The chloroplast dataset strongly supported the monophyly of *M. zhewanensis* (ML = 95%; BI = 1) and recovered it as sister to *M. caducifer* (ML = 96%; NJ = 0.91; BI = 1) (Fig 2). In contrast, the ITS dataset alone failed to resolve the relationship between *M. zhewanensis* and *M. caducifer*, with individuals of the two species intermingled and bootstrap support generally below 50% (S1–S3 Figs). The ILD test revealed significant incongruence between the ITS and chloroplast datasets (P = 0.001, based on 1,000 replicates), indicating conflicting phylogenetic signals between the two partitions. A combined analysis was therefore not performed. The incongruence between the chloroplast and ITS phylogenies indicates cytonuclear discordance, a phenomenon previously documented in *Mazus* [5,13,17], likely reflecting rapid diversification, incomplete lineage sorting, or chloroplast capture following historical hybridization. Marker choice also affects resolution: chloroplast regions, being maternally inherited and slower-evolving, typically resolve deeper nodes, whereas ITS often fails to recover recent divergences [5,13,17]. Despite these conflicts, the chloroplast dataset consistently recovered *M. zhewanensis* as a distinct, well-supported lineage.

**Fig 2:**
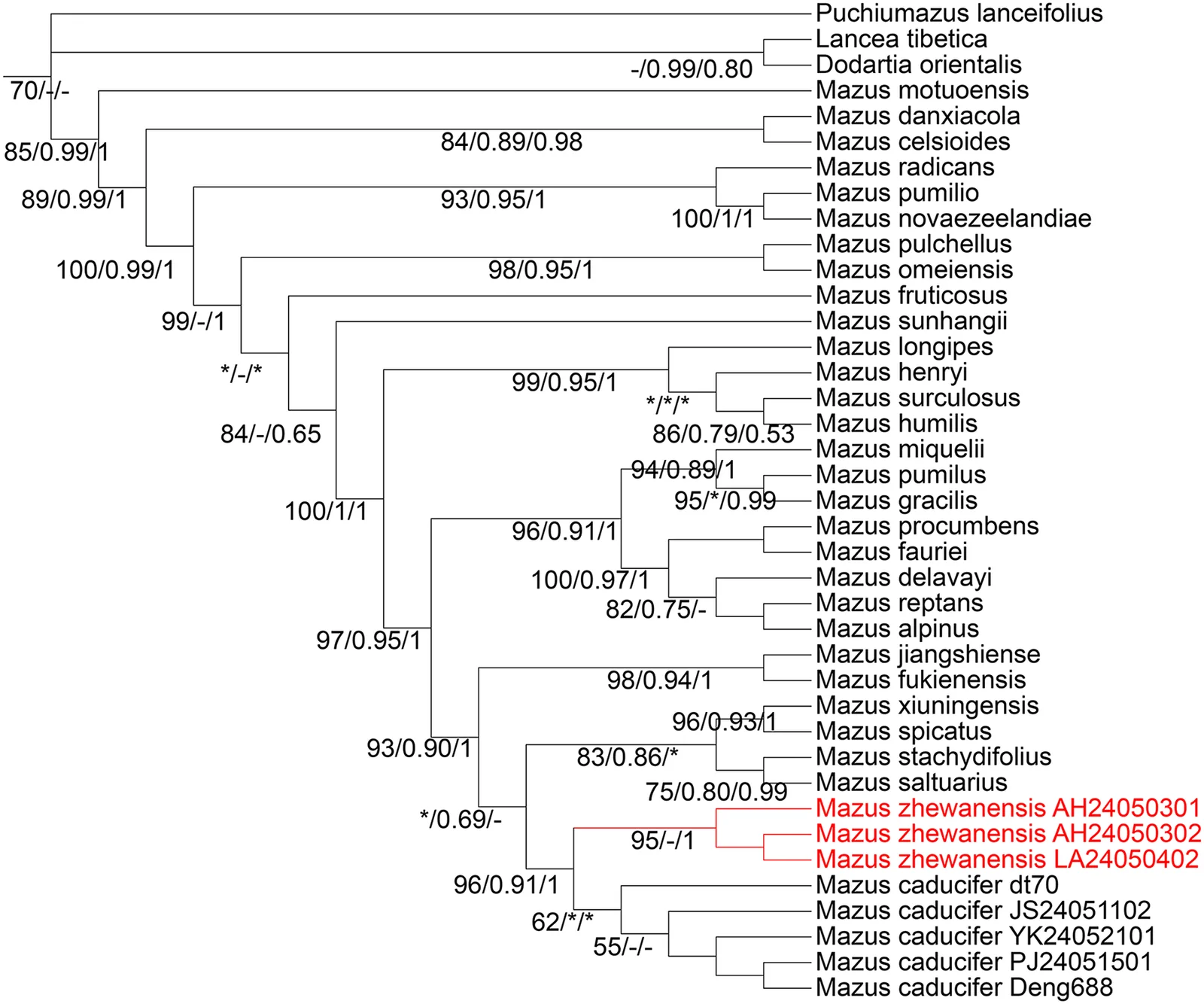
Maximum Likelihood (ML) phylogeny inferred from chloroplast DNA sequences (*rps16*, *rbcL*, and *trnL-trnF*), showing the phylogenetic position of *M. zhewanensis.* Support values at nodes are shown as ML bootstrap (%) / NJ bootstrap / Bayesian posterior probability. Asterisks (*) indicate ML bootstrap < 50%, NJ bootstrap < 0.5, or Bayesian posterior probability < 0.5. Dashes (-) indicate topological conflicts between the ML, NJ, and BI trees. The three accessions of *M. zhewanensis* are highlighted in red.

To confirm this result, we examined aligned chloroplast sequences of *M. zhewanensis* (n = 3) and *M. caducifer* (n = 5). Two fixed diagnostic sites were identified (Table 3). At alignment positions 950 and 1513, all *M. zhewanensis* individuals carry C, whereas all *M. caducifer* individuals carry T. The Kimura 2-parameter (K2P) genetic distance between the two species ranges from 0.0013 to 0.0020. These molecular data, together with the well-supported monophyly recovered in phylogenetic analyses, provide robust evidence for species delimitation.

**Table 3:** Fixed nucleotide differences between *M. zhewanensis* and *M. caducifer* in the combined chloroplast dataset.

Species	Collection no.	Alignment position 950	Alignment position 1513
*M. zhewanensis*	LA24050402	C	C
*M. zhewanensis*	AH24050301	C	C
*M. zhewanensis*	AH24050302	C	C
*M. caducifer*	dt70	T	T
*M. caducifer*	Deng688	T	T
*M. caducifer*	JS24051102	T	T
*M. caducifer*	PJ24051501	T	T
*M. caducifer*	YK24052101	T	T

The alignment position numbers refer to the combined chloroplast dataset (*rps*16 + *rbcL* + *trnL-trnF*).

### Taxonomic treatment

***Mazus zhewanensis* Z.H. Chen, W.Y. Xie et J.B. Pu, sp. nov. (**Figs 3–6)

**Fig 3:**
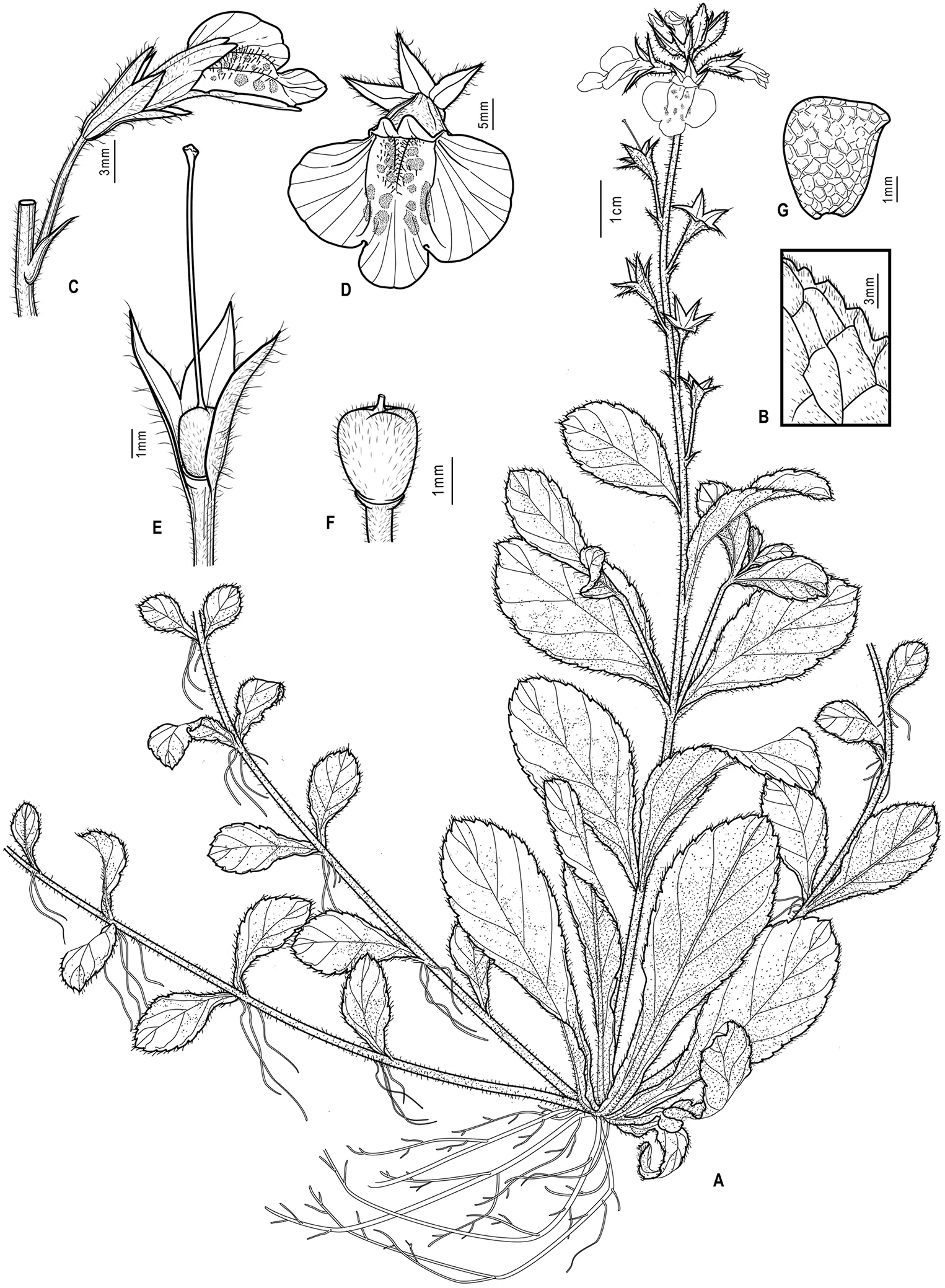
Line drawing of *M. zhewanensis* sp. nov. (A) Habit, (B) leaf margin, (C) flowering shoot, (D) corolla, (E) calyx and ovary, (F) capsule, (G) seed. Drawn by Yi Zhang.

**Fig 4:**
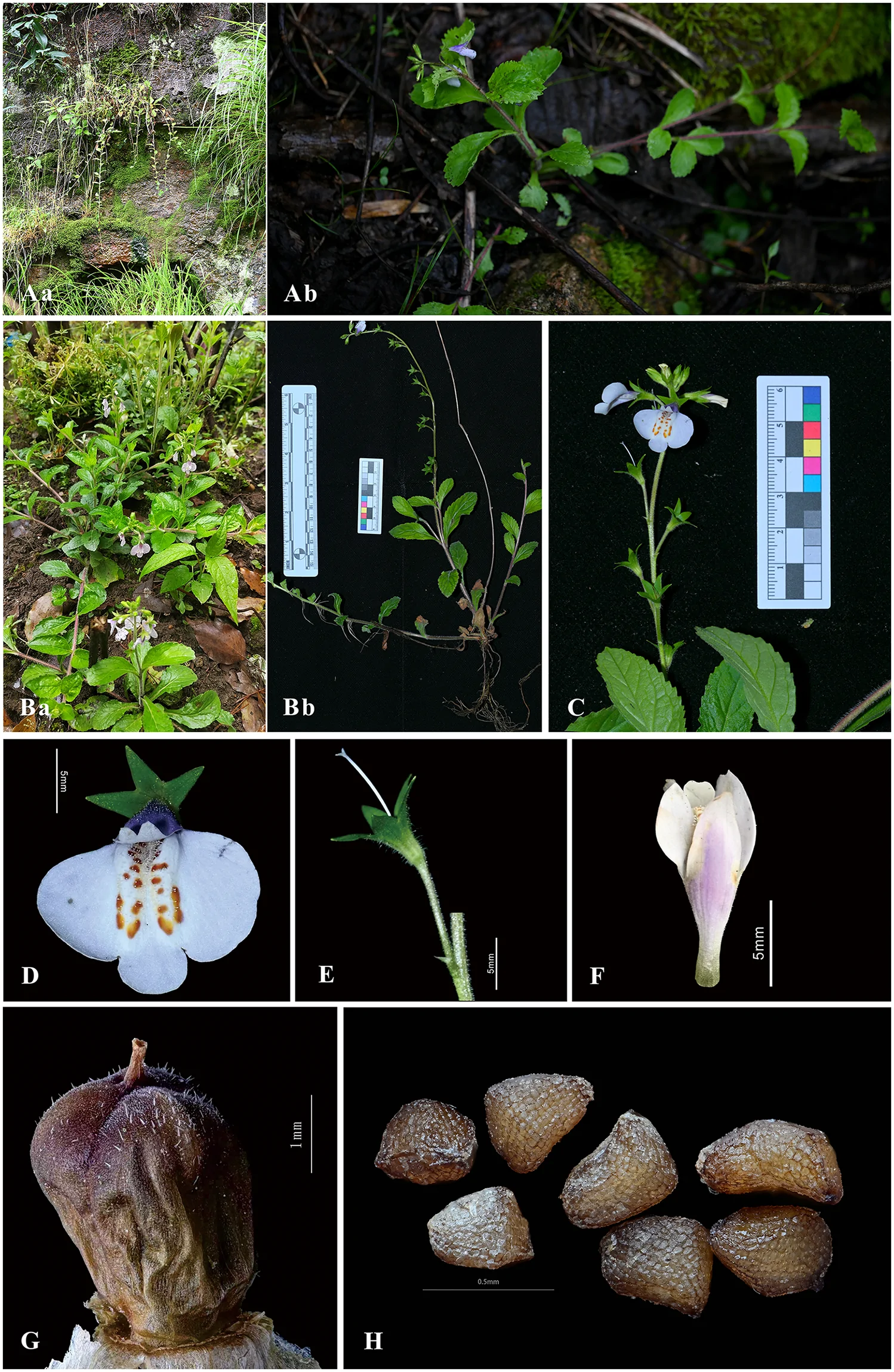
Photographs of *M. zhewanensis* sp. nov. in the field A. Habitat (Aa. Shady and moist steep slopes at forest edges; Ab. Shady and moist ravine forests); B. Plant (Ba. Erect stem and arching, non-rooting stolons at peak flowering; Bb. Erect stem and prostrate, rooted stolons at late anthesis); C. Inflorescence; D. Flower; E. Pedicel, calyx and style; F. Corolla; G. Fruit; H. Seeds.

**Fig 5:**
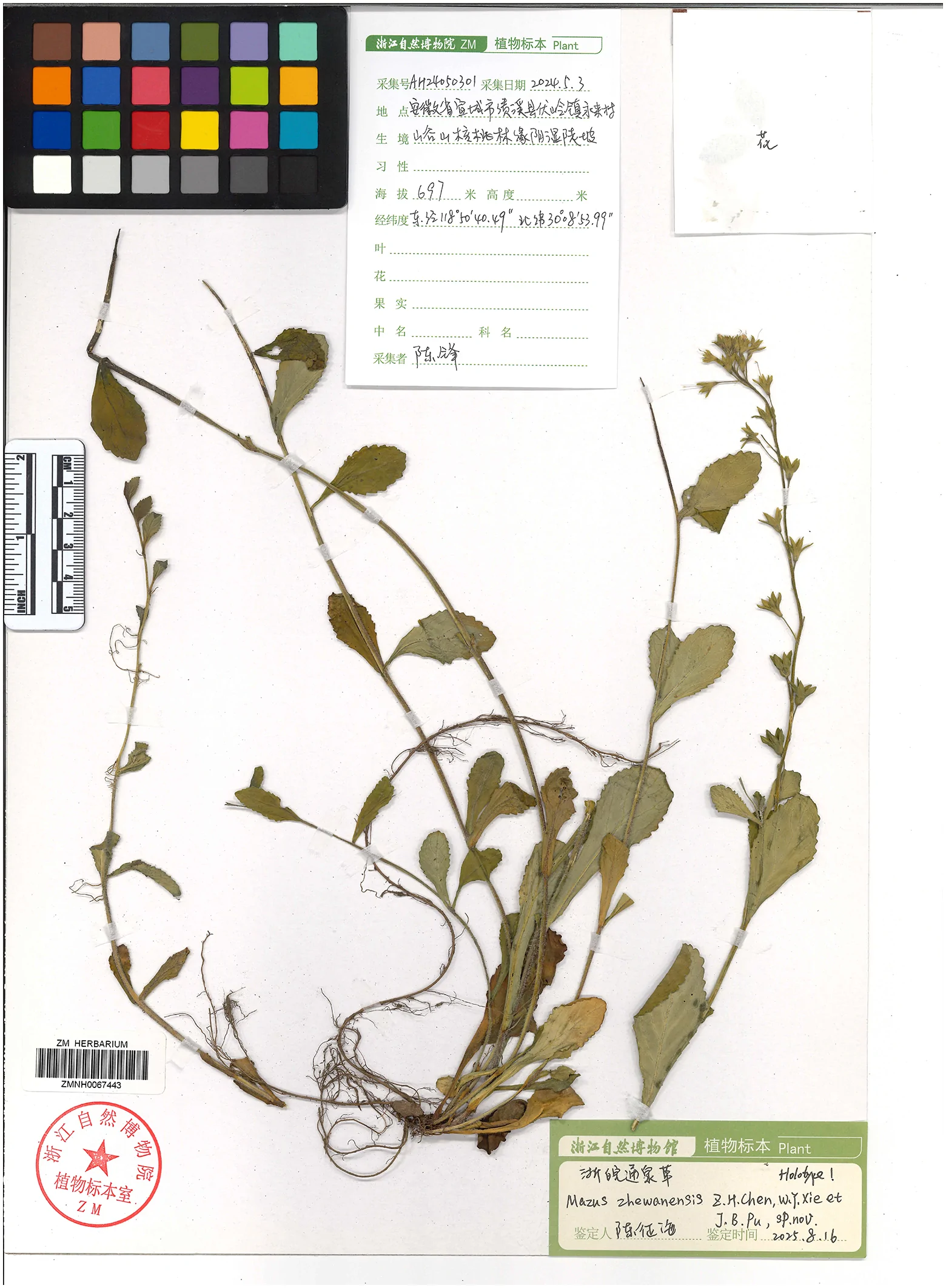
Holotype of *Mazus zhewanensis* sp. nov. (ZMNH0067443).

**Fig 6:**
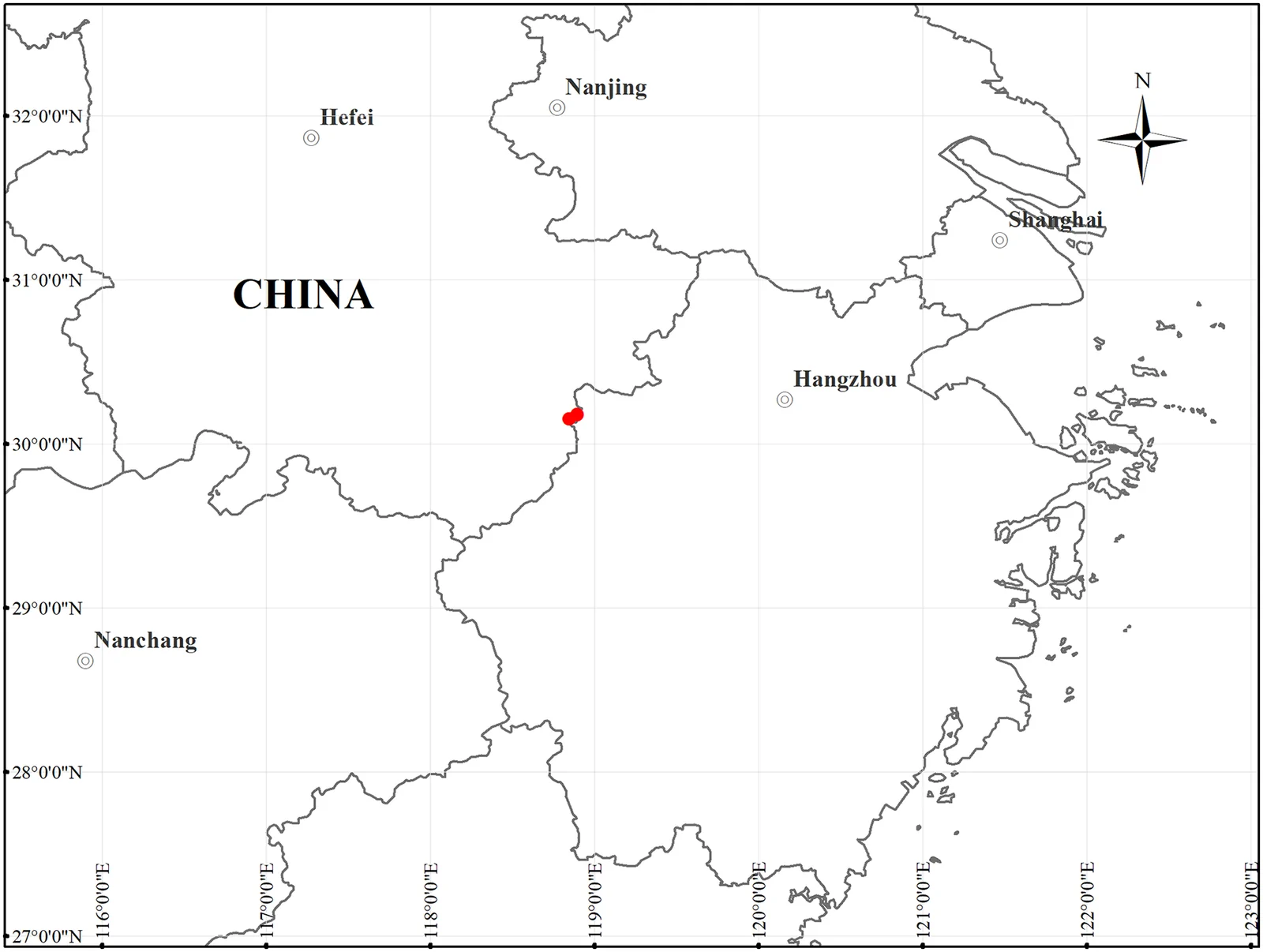
Geographic distribution of *M. zhewanensis* sp. nov. in Zhejiang & Anhui Province (red dots). Base map was sourced from the Standard Map Service of the Ministry of Natural Resources of China (审图号/Map Approval No.: GS(2024)0650). The base map is used without modification.

**[urn:lsid:ipni.org:names:77394106-1]**

Chinese name: **zhè-wǎn-tōng-quán-căo (浙皖通泉草)**.

Type: CHINA. Anhui Province, Xuancheng City, Jixi County, Fuling Town, Yonglai Village, on shaded and damp steep of forest margin of Carya cathayensis in valley, 118˚50′40.49″E, 30˚8′53.99″N, 697 m elev., 3 May 2024, *F. Chen AH24050301* (holotype: ZM [barcode: ZMNH0067443]; isotypes: PE [barcode: PE02687705], ZJMR [barcode: ZJMR015505], ZM [barcode: ZMNH0067444]).

Diagnosis. This new species is similar in appearance to *M. spicatus* Vaniot, but differs in its stolons 25–70 (–100) cm long (vs. 10–30 cm), leaf blade of erect stem elliptic with shallowly and densely serrate margin (vs. obovate to obovate-spatulate with incised-serrate margin), and corolla 1.4–2.0 cm long (vs. 0.8–1.2 cm). It is closely related to *M. caducifer* Hance, but differs in having long trailing stolons with adventitious roots produced at the nodes where they touch the ground (vs. no stolons and no adventitious roots at the nodes) and campanulate calyx (vs. infundibular calyx).

Description. ***Perennial herb***, taproot 1–2 cm long or inconspicuous; lateral roots slender, numerous, fascicled. ***Erect stems*** solitary, obtusely quadrangular, woody at the base with age, 6–38 cm in total height including the inflorescence, densely covered with spreading multicellular white villous; new branches often produced from leaf axils near the base of inflorescence late in flowering. ***Stolons*** arise from the base of erect stem or leaf axils near inflorescence base, somewhat slender, arching, pendent, or decumbent, 25–70(–100) cm long, nodes distant, producing adventitious roots where touching ground, internodes 3–13 cm, much longer than leaves. ***Basal leaves*** often withered at anthesis, subrosulate; blade ovate-elliptic, 1.7–4.5 × 1.3–2.0 cm; petiole 1.0–2.5 cm. Erect stem leaves 5–9, opposite or alternate in upper portion; blade elliptic, 2.5–4.0(–7.0) × 1.0–2.3(–3.0) cm; petiole 0.5–1.0 cm. ***Stolon leaves*** opposite or alternate in upper portion, basal 1–2 pairs often withered, the largest leaves located in middle-lower section; blade 2–3.8 × 1–2 cm, nearly sessile, leaves gradually smaller toward both ends. ***All leaves*** membranous, apex obtuse, base attenuate-cuneate and decurrent, margin shallowly and densely serrate, both surfaces sparsely white-strigose, veins beneath multicellular white villous. ***Inflorescence*** a terminal raceme on the main stem, 11–28 cm long, rachis densely multicellular white villous; flowers (6–)10–20 or more, loosely arranged; bracts narrowly linear-lanceolate, apex acuminate; pedicel 6–10 mm long, multicellular white villous. ***Calyx*** campanulate, 5–6 mm long at anthesis, 8–9 mm in fruit, ca. 4 mm in diameter, externally white-strigose and glandular-pubescent, glabrous within; veins 10, distinct, sparsely multicellular white villous; 5-lobed to middle, lobes lanceolate, nearly equal to or slightly longer than tube, margin ciliate. ***Corolla*** pale purple, 1.4–2.0 cm long, bilabiate; tube 6–8 mm; upper lip erect, 6–8 mm, apex 2-lobed, lobes narrowly triangular, entire; lower lip broad, 3-lobed, lateral lobes 6–10 mm, median lobe 8–12 mm, apex obtuse to emarginate, interior with 2 raised rugae at middle and throat, yellow-spotted, densely clavate-glandular. ***Stamens*** 4, the anterior pair longer than posterior; filaments coherent in pairs, adnate to corolla tube at middle-lower part, free portion of anterior filaments ca. 4.2 mm, posterior pair ca. 3 mm; anther cells 2, divaricate. ***Ovary*** obovoid, apex densely short-pilose intermixed with sparse, long hirsute hairs; style slender-cylindric; stigma deeply 2-lobed, lobes ovate, sparsely short-pilose along the margins. ***Capsule*** obovoid, ca. 2–3 mm in diameter, apex short-pilose intermixed with sparse, long hirsute hairs, dehiscing by 2-valves. ***Seeds*** small, numerous, surface finely reticulate.

Habitat and distribution. *M. zhewanensis* is primarily distributed in Lin’an District of Zhejiang Province and Jixi County of Anhui Province. It grows on shady, moist steep slopes at forest edges and moist ravine forests, at elevations of 650–1,350 m.

Phenology. Flowering from April to August, fruiting from June to October.

Etymology. The epithet “zhewanensis” is derived from the abbreviations of Zhejiang Province and Anhui Province, the type locality of the new species.

Conservation assessment. Based on currently available data, *M. zhewanensis* is confirmed from only two localities in Zhejiang and Anhui provinces, which likely represent two subpopulations. Preliminary estimates indicate a restricted Area of Occupancy (AOO) and a presumably small total population size. However, precise calculation of its Extent of Occurrence (EOO) and a comprehensive census of mature individuals are not yet available. Consequently, according to the IUCN Red List Categories and Criteria [46], this species is preliminarily assessed as Data Deficient (DD) due to the current uncertainties regarding its full distribution range and population parameters. Further field surveys are required to collect the necessary data for a definitive conservation status assessment.

Additional specimens examined (paratypes)**.** CHINA. Zhejiang Province, Hangzhou City, Lin’an District, Qingliangfeng Town, Maxiao, Langchuan Village, Taizijian, in wet place of the valley, 1,344 m elev., 118˚53'40.08"E, 30˚10'29.16"N, Z. H. Chen LA24050402 (ZM); the same locality and date, Zhexitianlu, in damp place by small ditch edge, Z. H. Chen LA24050403 (ZM). Anhui Province, Xuancheng City, Jixi County, Fuling Town, Yonglai Village, on shaded and damp steep of forest margin of Carya cathayensis in valley, 694 m elev., 118˚50'40.8"E, 30˚8'52.56"N, 21 Jun. 2023, Z.H. Chen et H.W. Zhang AH23062101, AH23062102 (ZM).

Key to the pubescent-fruited closely species of *Mazus* in China.

1 Plants without stolons, never producing adventitious roots at nodes  ........................2
1 Plants with stolons, producing adventitious roots at nodes where touching ground  .3
2 Stems erect; cauline leaves sessile; leaf blade oblong to oblanceolate; pedicel shorter than or nearly equaling calyx; corolla deep bluish-purple  ......................................................................................*M. stachydifolius*
2 Stems erect or ascending; cauline leaves with a winged petiole; leaf blade ovate-spatulate; pedicel equal to or longer than calyx; corolla pale purple  ........................................................................................................*M. caducifer*
3 Plants lacking a distinct erect stems; stolons slender and procumbent; leaf blade suborbicular or ovate, base truncate or broadly cuneate  ........................................................................................................................4
3 Plants with both erect stems and stolons; stolons somewhat slender, arching, procumbent, or decumbent; leaf blade elliptic, base cuneate  .......................................................................................................................5
4 Inflorescence terminal; upper lip of corolla with lobe apices irregularly dentate; calyx lobes nearly equal to tube  ..........................................................*M. xiuningensis*
4 Inflorescence terminal and axillary; upper lip of corolla with lobe apices entire; calyx lobes longer than tube  ...............................................................*M. jiangshiense*
5 Cauline leaves on erect stems obovate to obovate-spatulate, margin incised-serrate; corolla 0.8–1.2 cm long; stolons 10–30 cm long  ......................................*M. spicatus*
5 Cauline leaves on erect stems elliptic, margin shallowly and densely serrate; corolla 1.4–2.0 cm long; stolons 25–70(–100) cm long  ..................................*M. zhewanensis*

### Integrative taxonomy and species delimitation

We adopted an integrative taxonomic framework combining morphological, molecular phylogenetic, and ecological evidence for species delimitation. This framework follows contemporary standards emphasizing multiple independent lines of evidence, population-level sampling, and open data accessibility. Integration of these complementary datasets robustly supports *M. zhewanensis* as a distinct species and avoids taxonomic decisions based solely on minor morphological differences.

## Supporting information

S1 Table List of examined specimens of *Mazus* for morphological comparison. (XLSX)

S2 Table Kimura 2-parameter (K2P) genetic distances among all taxa analyzed. (XLSX)

S3 Table Fixed nucleotide diagnostic sites between *M. zhewanensis* and *M. caducifer.* (XLSX)

S1 Fig ITS ML tree. (PDF)

S2 Fig ITS NJ tree. (PDF)

S3 Fig ITS BI tree. (PDF)
