# Supplementary material for: Mazus zhewanensis (Mazaceae): A new species from Eastern China
Source: PLoS One. 2026 Oct 7;21(10):e0356991. doi: 10.1371/journal.pone.0356991 (PMC13645076; doi:10.1371/journal.pone.0356991)
Supplement: Supplementary file 1 [file pone.0356991.s004.pdf]

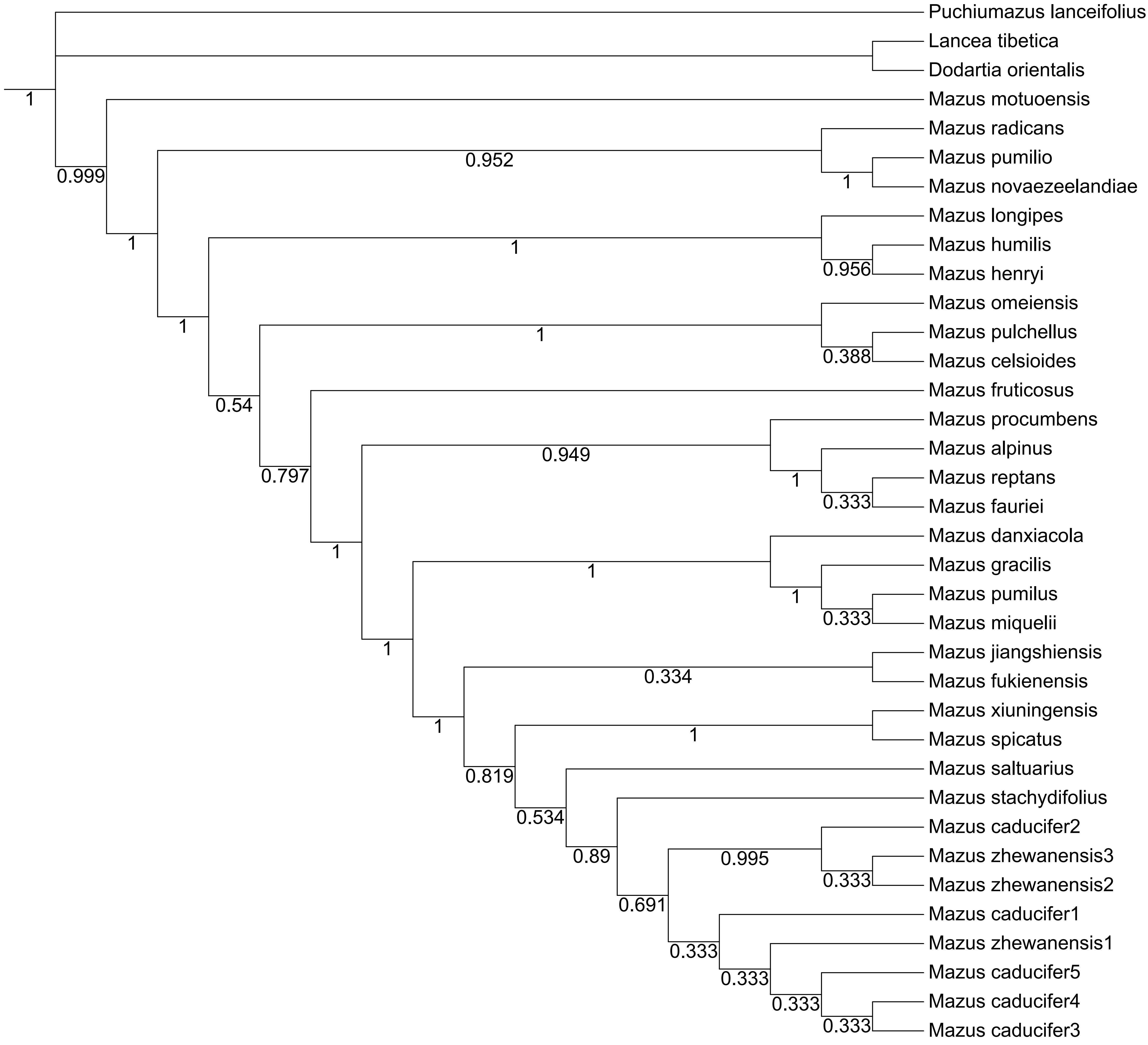
Puchiumazus lanceifolius
Lancea tibetica
Dodartia orientalis
Mazus motuoensis
Mazus radicans
Mazus pumilio
Mazus novaezeelandiae
Mazus longipes
Mazus humilis
Mazus henryi
Mazus omeiensis
Mazus pulchellus
Mazus celsioides
Mazus fruticosus
Mazus procumbens
Mazus alpinus
Mazus reptans
Mazus fauriei
Mazus danxiacola
Mazus gracilis
Mazus pumilus
Mazus miquelii
Mazus jiangshiensis
Mazus fukienensis
Mazus xiuningensis
Mazus spicatus
Mazus saltuarius
Mazus stachydifolius
Mazus caducifer2
Mazus zhewanensis3
Mazus zhewanensis2
Mazus caducifer1
Mazus zhewanensis1
Mazus caducifer5
Mazus caducifer4
Mazus caducifer3
1
0.999
1
0.952
1
1
1
0.956
1
1
0.388
0.54
0.797
0.949
1
0.333
1
1
1
1
0.333
1
0.334
1
0.819
0.534
0.89
0.995
0.333
0.691
0.333
0.333
0.333
0.333
