# Supplementary material for: Mazus zhewanensis (Mazaceae): A new species from Eastern China
Source: PLoS One. 2026 Oct 7;21(10):e0356991. doi: 10.1371/journal.pone.0356991 (PMC13645076; doi:10.1371/journal.pone.0356991)

PLOS One

Mazus zhewanensis (Mazaceae), a new species from Eastern China

--Manuscript Draft--

**Manuscript Number:**	PONE-D-26-09521
**Article Type:**	Research Article
**Full Title:**	Mazus zhewanensis (Mazaceae), a new species from Eastern China
**Short Title:**	Mazus zhewanensis, a new species from Eastern China
**Corresponding Author:**	Kelang Lou<br>Zhejiang Academy of Traditional Chinese Medicine<br>Hangzhou, CHINA
**Keywords:**	Mazus; molecular systematics; phylogeny; plastid genome; taxonomy
**Abstract:**	A new species of Mazus Lour. (Mazaceae), M. zhewanensis sp. nov., is described from East China. Morphologically, it is most similar to M. spicatus but is readily distinguished by its longer stolons [25–70(–100) cm], the elliptic leaves with shallowly and densely serrate margins on the erect stems, and a larger corolla (1.4–2.0 cm). Phylogenetic analyses based on maximum likelihood and Bayesian inference strongly support M. zhewanensis as an independent species. Additionally, a dichotomous key to M. zhewanensis and its pubescent-fruited species within the genus has been compiled, providing a basis for the identification of this new species.
**Order of Authors:**	Kelang Lou
	Wenyuan Xie
	Pan Xu
	Hongwei Zhang
	Feng Chen
	Junping Li
	Zhenghai Chen
	Weiqing Liang
**Additional Information:**	
**Question**	**Response**
**Financial Disclosure**<br><br>Enter a financial disclosure statement that describes the sources of funding for the work included in this submission. Review the submission guidelines for detailed requirements. View published research articles from *PLOS ONE* for specific examples.<br><br>This statement is required for submission and **will appear in the published article** if the submission is accepted. Please make sure it is accurate.	Yes

Powered by Editorial Manager® and ProduXion Manager® from Aries Systems Corporation

**Funded studies**<br>Enter a statement with the following details:<br>• Initials of the authors who received each award<br>• Grant numbers awarded to each author<br>• The full name of each funder<br>• URL of each funder website<br>• Did the sponsors or funders play any role in the study design, data collection and analysis, decision to publish, or preparation of the manuscript?<br><br>Did you receive funding for this work?	
Please add funding details.<br>as follow-up to "**Financial Disclosure**<br><br>Enter a financial disclosure statement that describes the sources of funding for the work included in this submission. Review the submission guidelines for detailed requirements. View published research articles from *PLOS ONE* for specific examples.<br><br>This statement is required for submission and **will appear in the published article** if the submission is accepted. Please make sure it is accurate.<br><br>**Funded studies**<br>Enter a statement with the following details:<br>• Initials of the authors who received each award<br>• Grant numbers awarded to each author<br>• The full name of each funder<br>• URL of each funder website<br>• Did the sponsors or funders play any role in the study design, data collection and analysis, decision to publish, or preparation of the manuscript?<br><br>Did you receive funding for this work?"	This study was financially supported by Major Central Government Budget Increases and Decreases (2060302), the Project of Survey, File and Codification of Wild Plants and the Second Edition of Flora of Zhejiang (335010-2015-0005)，the Zhejiang Key Discipline in Traditional Chinese Medicine for Pharmaceutical Botany (2024-XK-06), the Investigation and Research on National Key Protected Wild Plants in Lin'an District (231012), and the Research on the Classification of Doubtful Plants in Zhejiang Province (ZFSPLD2025-01).
Please select the country of your main research funder (please select carefully as in some cases this is used in fee calculation).	CHINA - CN

Powered by Editorial Manager® and ProduXion Manager® from Aries Systems Corporation

as follow-up to "**Financial Disclosure**<br><br>Enter a financial disclosure statement that describes the sources of funding for the work included in this submission. Review the submission guidelines for detailed requirements. View published research articles from *PLOS ONE* for specific examples.<br><br>This statement is required for submission and **will appear in the published article** if the submission is accepted. Please make sure it is accurate.<br><br>**Funded studies**<br>Enter a statement with the following details:<br>• Initials of the authors who received each award<br>• Grant numbers awarded to each author<br>• The full name of each funder<br>• URL of each funder website<br>• Did the sponsors or funders play any role in the study design, data collection and analysis, decision to publish, or preparation of the manuscript?<br><br>Did you receive funding for this work?"	
**Competing Interests**<br><br>Use the instructions below to enter a competing interest statement for this submission. On behalf of all authors, disclose any competing interests that could be perceived to bias this work—acknowledging all financial support and any other relevant financial or non-financial competing interests.<br><br>This statement is required for submission and **will appear in the published article** if the submission is accepted. Please make sure it is accurate and that any funding sources listed in your Funding Information later in the submission form are also declared in your Financial Disclosure statement.	The authors declare no conflict of interest.

Powered by Editorial Manager® and ProduXion Manager® from Aries Systems Corporation

View published research articles from *PLOS ONE* for specific examples.<br><br>**NO authors have competing interests**<br><br>Enter: *The authors have declared that no competing interests exist.*<br><br>**Authors with competing interests**<br><br>Enter competing interest details beginning with this statement:<br><br>*I have read the journal's policy and the authors of this manuscript have the following competing interests: [insert competing interests here]*<br><br>* typeset	
**Ethics Statement**<br><br>Enter an ethics statement for this submission. This statement is required if the study involved:<br><br>• Human participants<br>• Human specimens or tissue<br>• Vertebrate animals or cephalopods<br>• Vertebrate embryos or tissues<br>• Field research<br><br>Write "N/A" if the submission does not require an ethics statement.<br><br>General guidance is provided below. Consult the submission guidelines for detailed instructions. **Make sure that all information entered here is included in the Methods section of the manuscript.**	No ethical statement was reported.

**Format for specific study types**<br><br>**Human Subject Research (involving human participants and/or tissue)**<br>• Give the name of the institutional review board or ethics committee that approved the study<br>• Include the approval number and/or a statement indicating approval of this research<br>• Indicate the form of consent obtained (written/oral) or the reason that consent was not obtained (e.g. the data were analyzed anonymously)<br><br>**Animal Research (involving vertebrate animals, embryos or tissues)**<br>• Provide the name of the Institutional Animal Care and Use Committee (IACUC) or other relevant ethics board that reviewed the study protocol, and indicate whether they approved this research or granted a formal waiver of ethical approval<br>• Include an approval number if one was obtained<br>• If the study involved *non-human primates*, add *additional details* about animal welfare and steps taken to ameliorate suffering<br>• If anesthesia, euthanasia, or any kind of animal sacrifice is part of the study, include briefly which substances and/or methods were applied<br><br>**Field Research**<br><br>Include the following details if this study involves the collection of plant, animal, or other materials from a natural setting:<br>• Field permit number<br>• Name of the institution or relevant body that granted permission	
**Data Availability**<br><br>Authors are required to make all data underlying the findings described fully available, without restriction, and from the time of publication. PLOS allows rare exceptions to address legal and ethical concerns. See the PLOS Data Policy and FAQ for detailed information.	Yes - all data are fully available without restriction

A Data Availability Statement describing where the data can be found is required at submission. Your answers to this question constitute the Data Availability Statement and **will be published in the article**, if accepted.<br><br>**Important:** Stating 'data available on request from the author' is not sufficient. If your data are only available upon request, select 'No' for the first question and explain your exceptional situation in the text box.<br><br>Do the authors confirm that all data underlying the findings described in their manuscript are fully available without restriction?	
**Describe where the data may be found in full sentences. If you are copying our sample text, replace any instances of XXX with the appropriate details.**<br><br>• If the data are **held or will be held in a public repository**, include URLs, accession numbers or DOIs. If this information will only be available after acceptance, indicate this by ticking the box below. For example: *All XXX files are available from the XXX database (accession number(s) XXX, XXX.).*<br>• If the data are all contained **within the manuscript and/or Supporting Information files**, enter the following: *All relevant data are within the manuscript and its Supporting Information files.*<br>• If neither of these applies but you are able to provide **details of access elsewhere**, with or without limitations, please do so. For example:<br><br>*Data cannot be shared publicly because of [XXX]. Data are available from the XXX Institutional Data Access / Ethics Committee (contact via XXX) for researchers who meet the criteria for access to confidential data.*<br><br>*The data underlying the results presented in the study are available from (include the name of the third party*	All sequence data generated in this study are available from the NCBI GenBank database. The accession numbers are listed in Table 1 of the manuscript.

and contact information or URL).<br>• This text is appropriate if the data are owned by a third party and authors do not have permission to share the data.<br><br>* typeset	
Additional data availability information:	

Cover Letter

Click here to access/download;Manuscript;renamed_3234c.docx

Mazus zhewanensis (Mazaceae), a new species from Eastern China

Ke-Lang Lou[1,2¶], Wen-Yuan Xie[4¶], Pan Xu[1,2], Hong-Wei Zhang[5], Feng Chen[4], Jun-Ping Li[6], Zheng-Hai Chen[4,7*], Wei-Qing Liang[1,2,3*]

[1] *Zhejiang Academy of Traditional Chinese Medicine, Hangzhou, Zhejiang, China*

[2] *Tongde Hospital of Zhejiang Province, Hangzhou, Zhejiang, China*

[3] *State Key Laboratory for Quality Assurance and Sustainable Use of Dao-di Herbs, Beijing, China*

[4] *Zhejiang Forest Resources Monitoring Center, Hangzhou, Zhejiang, China*

[5] *Administration of Zhejiang Qingliangfeng National Natural Reserve, Hangzhou, Zhejiang, China*

[6] *Yongkang Forestry Bureau, Yongkang, China*

[7] *Zhejiang Forestry Survey Planning and Design Co., Ltd, Hangzhou, Zhejiang, China*

* Corresponding author

E-mail: jxlwq22@163.com (WL); zhchen1963@163.com (ZC)

¶ These authors contributed equally to this work.

Abstract

A new species of *Mazus* Lour. (Mazaceae), *M. zhewanensis* **sp. nov.**, is described from East China. Morphologically, it is most similar to *M. spicatus* but is readily distinguished by its longer stolons [25–70(–100) cm], the elliptic leaves with shallowly and densely serrate margins on the erect stems, and a larger corolla (1.4–2.0 cm). Phylogenetic analyses based on maximum likelihood and Bayesian inference strongly support *M. zhewanensis* as an independent species. Additionally, a dichotomous key to *M. zhewanensis* and its pubescent-fruited species within the genus has been compiled, providing a basis for the identification of this new species.

Introduction

The genus *Mazus* Lour. (Mazaceae), as circumscribed by POWO [1], comprises approximately 45 accepted species distributed primarily across Asia and Australasia [2-4]. In China, the genus is widespread, with its diversity center located in the southwestern and central regions [2,5].

Eastern China represents a significant area for the genus, with 13 species currently documented. These species exhibit two distinct distribution patterns. (1) species whose primary ranges are centered within Eastern China, and (2) species for which the region constitutes a marginal part of a broader distribution extending into southwestern China or central China or other parts of East Asia [5-14]. Historically, most species recorded from Eastern China belonged to the latter group. Prior to the

description of *M. xiuningensis* X.H. Guo & X.L. Liu [15], the region was known mainly for widespread taxa such as *M. stachydifolius* (Turcz.) Maxim. [16], *M. caducifer* Hance [17], *M. pumilus* (Burm. f.) Steenis [18], *M. miquelii* Makino [19], and *M. gracilis* Hemsl. [20]. *M. spicatus* Vaniot [21], sparsely recorded only within Anhui Province [7], also belongs to this marginal-distribution category.

Since the publication of the Flora of China, however, a series of newly described species have revealed Eastern China as an important center of endemism for *Mazus*. These include Fujian endemics such as *M. fukienensis* P.C. Tsoong [22], *M. wanmuliensis* M. Qian & L.B. Geng [23], and *M. jiangshiensis* Y.B. Chen, Xin Y. Chen & Liang Ma [24]; the Anhui endemic *M. xiuningensis* and *M. apicaliracemosus* X.H. Guo, X.P. Zhang et J.L. Xu [25]; and species with distributions spanning adjacent provinces, such as *M. jingtaiensis* Z.H. Chen, W.Y. Xie & X.W. Zhang (Zhejiang/Fujian; [26]) and *M. saltuarius* Hand.-Mazz. [27] (Zhejiang/Jiangxi; [12, 14, 28]). Together, these findings highlight Eastern China as a notable center of diversification and endemism for *Mazus*.

During recent field investigations of wild plant resources in Zhejiang and Anhui, we discovered an unknown taxon belonging to *Mazus* sect. *Trichogynus*, characterized by a pubescent ovary, but is distinguished from all known congeners in the section by its numerous and elongated stolons. Detailed morphological examination, field observations, comparison with type material, and phylogenetic analyses based on chloroplast and nuclear DNA markers consistently supported its separation from all

described taxa, particularly from the morphologically similar *M. spicatus* and *M. caducifer*.

Here, we describe this taxon as *Mazus zhewanensis* sp. nov., provide diagnostic characters distinguishing it from related species, and present molecular evidence supporting its species status. A key to the pubescent-fruited species of *Mazus* in China is also provided.

Material and methods

Taxon sampling

Fresh leaves from three populations were collected for DNA extraction. The leaves were placed in non-woven fabric bags and dehydrated in sealed bags with color-indicator silica gel. Voucher specimens from each population were deposited in the Herbarium of Medicinal Resources at the Zhejiang Academy of Traditional Chinese Medicine (ZJMR); the Zhejiang Museum of Natural History (ZM); and the Institute of Botany, Chinese Academy of Sciences (PE).

DNA extraction, PCR amplification, and sequencing

To elucidate the phylogenetic relationships and taxonomic placement of the proposed new species within Mazus, we conducted phylogenetic analyses based on three chloroplast DNA regions (rbcL, rps16, and trnL-trnF) and the nuclear ribosomal internal transcribed spacer (ITS). Polymerase chain reaction (PCR) amplification were

performed using the following primers: rbcL, RH1/Z1352RC [29]; rps16, rps16-2F/rps16-R3 [30]; trnL-trnF, the c/f primers [31]; and ITS, ITS1/ITS4 [32]. Laboratory protocols and downstream data processing followed the workflow outlined in Chen et al. [24].

Phylogenetic analyses

To determine the phylogenetic position of the newly discovered species, sequences data for rbcL, rps16, and trnL-trnF and ITS were obtained from *Mazus* species available in GenBank. *Dodartia orientalis* L. [33] and *Lancea tibetica* Hook.f. & Thomson [34] were chosen as outgroups following Li et al. [35]. Accession numbers for all taxa are listed in Table 1. Phylogenetic analyses were conducted using both chloroplast and ITS datasets. Maximum Likelihood (ML) analyses were performed in IQ-TREE 3.0.1 [36], with the best-fit substitution models selected by the built-in ModelFinder [37] and node support assessed using the Ultrafast Bootstrap [38] method with 1,000 replicates. Bayesian Inference (BI) was conducted using MrBayes [39] with Markov Chain Monte Carlo (MCMC) chains run for sufficient generations to ensure convergence. The congruent results from both methods were used to infer the evolutionary relationships among the species.

Table 1. Taxon and GenBank accession numbers of the specimens.

Taxa	rbcL	rps16	trnL-trnF	ITS
Dodartia orientalis L.	JQ342984	JQ342982	JQ342981	JQ342980
Lancea tibetica Hook.f. & Thomson	MF786661	FJ172699	FJ172685	FJ172736
M. alpinus Masam.	KX783481	KX783501	KX783520	MK192641
M. caducifer Hance	KX783477	KX783497	MK266455	MK192664
M. caducifer Hance	KX783487	KX783506	KX783526	MK192659
M. celsioides Hand.-Mazz.	KX783486	MK266366	KX783525	MT703875
M. danxiacola Bo Li & B.Chen	ON323565	ON323567	ON323569	ON286711
M. delavayi Bonati	KX783482	KX783502	KX783521	/
M. fauriei Bonati	KX783479	KX783499	KX783518	MK192640
M. fruticosus Bo Li, D.G.Zhang & C.L.Xiang	/	KX783490	KX783509	MK192660
M. fukienensis P.C.Tsoong	MT712054	MT712059	MT712064	MT707248
M. gracilis Hemsl.	FJ172729	FJ172701	FJ172687	FJ172738
M. henryi P.C.Tsoong	MT712055	MT712060	MT712065	MT707246
M. *humilis* Hand.-Mazz.	PQ824921	MK266401	MK266458	MK192667
M. *jiangshiensis* Y.B.Chen, Xin Y. Chen & Liang Ma	OP616018	OP616019	OP616020	OP605381
M. longipes Bonati	KX783474	KX783494	KX783513	MK192652
M. miquelii Makino	KX783475	KX783495	MK266423	MK192637
M. motuoensis W.B.Ju, Bo Xu bis & X.F.Gao	OQ383431	/	OQ383430	OP720888
M. novaezeelandiae W.R.Barker	KX783469	KX783489	KX783508	MK192676
M. omeiensis H.L.Li	KX807209	KX807203	KX807208	MK192636
M. procumbens Hemsl.	KX783478	KX783498	KX783517	MK192647
M. pulchellus Hemsl.	KX783472	KX783492	KX783511	MK192638
M. pumilio R.Br.	KX783468	KX783488	KX783507	MK192671
M. pumilus (Burm.f.) Steenis	FJ172728	FJ172700	FJ172686	FJ172737
M. radicans Cheeseman	/	MK266404	MK266461	MK192669
M. reptans N.E.	HQ384872	HQ385147	AF479004	AF478940
M. saltuarius Hand.-Mazz.	MT712057	MT712062	MT712068	MT707245
M. spicatus Vaniot	FJ172730	FJ172703	MK266471	FJ172740
M. stachydifolius (Turcz.) Maxim.	EU348860	AJ609167	MT712070	MT703903
M. sunhangii D.G.Zhang & T.Deng	KX783484	KX783504	KX783523	/
M. surculosus D.Don	KX783473	KX783493	KX783512	/
M. xiuningensis X.H.Guo & X.L.Liu	MT712058	MT712063	MT712071	MT707247
M. zhewanensis Z.H. Chen, W.Y Xie et J.B. Pu	PX578853*	PX578856*	PX578859*	PX607764*
M. zhewanensis Z.H. Chen, W.Y Xie et J.B. Pu	PX578854*	PX578857*	PX578860*	PX607765*
M. zhewanensis Z.H. Chen, W.Y Xie et J.B. Pu	PX578855*	PX578858*	PX578861*	PX607766*

*=self-acquired in this study; the others are from GenBank. /=represents data deficient.

Results and discussion

Morphological comparison

Based on the combination of an erect stem, exceptionally long stolons, elliptic leaves with shallowly and densely serrate margins on the erect stem, and a relatively large corolla, *M. zhewanensis* is readily distinguished from other pubescent-fruited species of *Mazus* in China. As shown in the provided key and comparative morphology (Table 2), it most resembles *M. spicatus*, but it differs decisively in the characters listed above. Furthermore, *M. zhewanensis* is readily distinguished from stoloniferous species lacking a distinct erect stem (e.g., *M. xiuningensis*, *M. jiangshiensis*) by its well-developed erect stem and corresponding leaf morphology.

Table 2. Comparison between *M. zhewanensis* and similar taxa.

Character		***M. zhewanensis***[$]	***M. spicatus***[α]	***M. xiuningensis***[β]	***M. jiangshiensis***[γ]
Basal Leaves		Often withering at anthesis, subrosulate; ~~blade~~ ovate-elliptic 1.7–4.5 × 1.3–2.0 cm, apex obtuse; petiole: 1.0–2.5 cm.	Few and early withering, apex caudate.	Orbicular, ovate, or elliptic, 1–6 × 0.8–4.0 cm, apex rounded or dentate-acute, margin irregularly sinuate-serrate; petiole: 1.5–6.5 cm.	Several to numerous, often deciduous.
Erect Stem	Stem	Erect, robust, woody at base, 6–38 cm tall (including inflorescence), densely white-villous; new branches often from leaf axils near inflorescence base late in flowering.	Erect, slender, stiff, woody at base, 10–30 cm high, white or pale rusty tomentose.	Diffuse, inconspicuous.	Diffuse, inconspicuous.
	Leaf	~~Blade~~ elliptic, 2.5–4.0 (-7.0) × 1.0-2.3 (-3.0) cm, apex obtuse, margin shallowly and densely serrate; petiole: 0.5–1.0 cm.	Obovate to obovate-spatulate, 1–4 cm, margin incised-serrate; petiole: up to 1 cm on lower leaves, reducing upwards to nearly sessile.	/	/
Stolon Stem	Stem	Arising from base	Decumbent-ascending,	Profusely	Profusely

		of erect stem or leaf axils near inflorescence base, somewhat slender, arching, procumbent, or decumbent, 25–70(–100) cm, nodes distant, rooting and producing new leaf tufts late in flowering, internodes 3–13 cm, much longer than leaves.	slender, 10–30 cm, nodal rooting.	branched, slender and procumbent, densely pubescent, nodal rooting.	branched, slender base woody, creeping to 50 cm, nodal rooting.
	Leaf	Largest in the middle-lower section; blade: 2.0–3.8 × 1.0–2.0 cm, apex obtuse, margin shallowly and densely serrate, nearly sessile.	Same as leaves on the erect stem.	Same as basal leaves, but blade size and petiole length gradually reduce upwards.	Subrounded or oblong, 2.5–5.5 × 2–4 cm (including petiole), larger at stem base, apex acute to obtuse, base truncate and tapering, margin coarsely crenate; petiole: 0.5–2.5 cm.
Inflorescence		11–28 cm long, with (6–)10–20 flowers; pedicel: 6–10 mm.	6.5–20 cm long.	With 3–10 flowers; pedicel: 0.5–6 mm.	To 17.0 cm or longer, multiflowered to 20; pedicel: ca. 6 mm in fruit.
Calyx		Campanulate, 5–6 mm at anthesis, accrescent to 8–9 mm in fruit, sparsely white-strigose and glandular-pubescent externally; veins distinct; lobes lanceolate, nearly equal to or slightly longer than tube.	Campanulate, 5–8 mm in fruit, diameter <1 cm; veins slightly prominent; margin short-ciliate; lobes triangular-lanceolate, acute, nearly equal to tube.	Broadly campanulate, 3–5 mm at anthesis, 3–8 mm in fruit, villous externally; lobes ovate, shorter than the tube	Infundibular (funnelform), 3.0–4.0 mm, slightly accrescent in fruit, villous on both surfaces; lobes 5, lanceolate, acute, longer than tube.
Corolla		1.4-2.0 cm long; upper lip 6–8 mm, apex 2-lobed, lobes narrowly triangular, entire; lower lip: lateral lobes 6–10 mm, middle lobe 8–12 mm	0.8–1.2 cm long; upper lip apex bilobed, lobes narrowly acute, entire; lower lip with lateral lobes orbicular, entire; middle lobe slightly narrower and longer, apex emarginate.	1.3–1.7 cm long; upper lip 4–6 mm, apex bilobed, lobe apices irregularly dentate; lower lip with lateral lobes 5.5–7.5 mm and middle lobe 7–9 mm.lobe 7–9 mm	0.9–1.1 cm long; upper lip bilobed, lobes lanceolate, acute, entire; lower lip trilobed, middle lobe oblong, smaller and narrower than the broadly ovate lateral lobes.
Filaments		Anterior pair ca. 4.2 mm, posterior pair ca. 3.0 mm.	/	Anterior pair 3.5–4.5 mm, posterior pair 2.5–3 mm.	Anterior pair longer , curved.
Ovary		Densely short-pilose intermixed with sparse long-hirsute hairs.	Hirsute.	Densely clavate-glandular hairy.	Villous.
Phenology		Flowering: April – August; Fruiting: June – October.	Flowering: May–June; Fruiting: July–August.	Flowering: June–July; Fruiting: August–September.	Flowering: June–July; Fruiting: August–September.
Distribution		Northwestern Zhejiang (Lin'an),	Hunan, Hubei, Guangxi, Guizhou,	Southeastern Anhui (Xiuning).	Northwest Fujian (Shaowu).

	Southeastern Anhui (Jixi).	Sichuan, and Southern Shaanxi.

Based on Vaniot 1905α, Guo and Liu 1990β, Chen et al.γ; and own measurements at ZJMR $, "/" represents data deficient.

Molecular phylogenetic relationship

Both ML and BI analyses yielded highly congruent topologies (Fig 1). The three accessions of the proposed new species, *M. zhewanensis*, formed a strongly supported monophyletic clade (BP = 81%, PP = 0.99), confirming their conspecific status. This clade was consistently recovered as sister to *M. caducifer* (BP = 93%, PP = 1.00). These high support values indicate that *M. zhewanensis* is a phylogenetically distinct lineage, closely related to but evolutionarily independent from *M. caducifer*. This molecular evidence therefore provides robust support for the recognition of *M. zhewanensis* as a new species.

Figure 1. A Maximum Likelihood phylogeny, reconstructed from combined ITS and chloroplast DNA sequences, was used to determine the phylogenetic position of *M. zhewanensis*. Nodes are labeled with support values (BP/PP for bootstrap and posterior probability) only if BP ≥ 50% and PP ≥ 0.50. Asterisks (*) indicate BP < 50%, and dashes (-) mark topological conflict between ML and BI trees. The accessions of the new species are highlighted in red.

Taxonomic treatment

***Mazus zhewanensis* Z.H. Chen, W.Y. Xie et J.B. Pu, sp. nov. (**Figs 2–4**)**

Figure 2. Line drawing of *M. zhewanensis* sp. nov. (A) habit, (B) leaf margin, (C) flowering shoot, (D) corolla, (E) calyx and ovary, (F) capsule, (G) seed. Drawn by Zhang Yi.

Figure 3. Photographs of *M. zhewanensis* sp. nov. in the field A. Habitat (Aa. Shady and moist steep slopes at forest edges; Ab. Shady and moist ravine forests); B. Plant (Ba. Erect stem and arching, non-rooting stolons at peak flowering; Bb. Erect stem and prostrate, rooted stolons at late anthesis); C. Inflorescence; D. Flower; E. Pedicel, calyx and style; F. Corolla; G. Fruit; H. Seeds.

Figure 4. Geographic distribution of *M. zhewanensis* sp. nov. in Zhejiang & Anhui Province (red dots).

Chinese name: zhè-wǎn-tōng-quán-cǎo (浙皖通泉草).

Type: CHINA. Anhui Province, Xuancheng City, Jixi County, Fuling Town, Yonglai Village, on shaded and damp steep of forest margin of Carya cathayensis in valley, 118°50′40.49″E, 30°8′53.99″N, ~~alt.~~ 697 m ~~a.s.l.~~, 3 May 2024, *F. Chen AH24050301* (holotype: ZM; isotypes: PE, ZJMR, ZM).

Diagnosis. This new species is similar in appearance to *M. spicatus* Vaniot, but differs in its stolon 25–70 (–100) cm long (vs. 10–30 cm), leaf blade of erect stem elliptic with shallowly and densely serrate margin (vs. obovate to obovate-spatulate

with incised-serrate margin), and corolla 1.4–2.0 cm long (vs. 0.8–1.2 cm). It is closely related to *M. caducifer* Hance, but differs in having long trailing stolons with adventitious roots produced at the nodes where they touch the ground (vs. no stolons and no adventitious roots at the nodes) and campanulate calyx (vs. infundibular calyx).

Description. ***Perennial herb***, taproot 1–2 cm long or inconspicuous; lateral ~~and fibrous~~ roots slender, numerous, fascicled. ***Erect stems*** solitary, obtusely quadrangular, woody at the base with age, 6–38 cm in total height including the inflorescence, densely covered with spreading multicellular white villous; new branches often produced from leaf axils near the base of inflorescence late in flowering. ***Stolons*** arise from the base of erect stem or leaf axils near inflorescence base, somewhat slender, arching, pendent, or decumbent, 25–70(–100) cm long, nodes distant, producing adventitious roots where touching ground, internodes 3–13 cm, much longer than leaves. ***Basal leaves*** often withered at anthesis, subrosulate; blade ovate-elliptic, 1.7–4.5 × 1.3–2.0 cm; petiole 1.0–2.5 cm. Erect stem leaves 5–9, opposite or alternate in upper portion; blade elliptic, 2.5–4.0(–7.0) × 1.0–2.3(–3.0) cm; petiole 0.5–1.0 cm. ***Stolon leaves*** opposite or alternate in upper portion, basal 1–2 pairs often withered, the largest leaves located in middle-lower section; blade 2–3.8 × 1–2 cm, nearly sessile, leaves gradually smaller toward both ends. ***All leaves*** membranous, apex obtuse, base attenuate-cuneate and decurrent, margin shallowly and densely serrate, both surfaces sparsely white-strigose, veins beneath multicellular white villous. ***Inflorescence*** a terminal raceme on the main stem, 11–28 cm long, rachis densely

multicellular white villous; flowers (6–)10–20 or more, loosely arranged; bracts narrowly linear-lanceolate, apex acuminate; pedicel 6–10 mm long, ~~longer than calyx~~, multicellular white villous. ***Calyx*** campanulate, 5–6 mm long at anthesis, 8–9 mm in fruit, ca. 4 mm in diameter, externally white-strigose and glandular-pubescent, glabrous within; veins 10, distinct, sparsely multicellular white villous; 5-lobed to middle, lobes lanceolate, nearly equal to or slightly longer than tube, margin ciliate. Corolla pale purple, 1.4–2.0 cm long, bilabiate; tube 6–8 mm; upper lip erect, 6–8 mm, apex 2-lobed, lobes narrowly triangular, entire; lower lip broad, 3-lobed, lateral lobes 6–10 mm, median lobe 8–12 mm, apex obtuse to emarginate, interior with 2 raised rugae at middle and throat, yellow-spotted, densely clavate-glandular. ***Stamens*** 4, the anterior pair longer than posterior; filaments coherent in pairs, adnate to corolla tube at middle-lower part, free portion of anterior filaments ca. 4.2 mm, posterior pair ca. 3 mm; anther cells 2, divaricate. ***Ovary*** obovoid, apex densely short-pilose intermixed with sparse, long hirsute hairs; style slender-cylindric; stigma deeply 2-lobed, lobes ovate, sparsely short-pilose along the margins. ***Capsule*** obovoid, ca. 2–3 mm in diameter, apex short-pilose intermixed with sparse, long hirsute hairs, dehiscing by ~~2 valves~~. ***Seeds*** small, ~~very~~ numerous, surface finely reticulate.

Habitat and distribution. *M. zhewanensis* is primarily distributed in Lin'an District of Zhejiang Province and Jixi County of Anhui Province. It grows on shady, moist steep slopes at forest edges and moist ravine forests, at elevations of 650–1,350 m ~~a.s.l.~~.

Phenology. Flowering from April to August, fruiting from June to October.

Etymology. The epithet "zhewanensis" is derived from the abbreviations of Zhejiang Province and Anhui Province, the type locality of the new species.

Conservation assessment. Based on currently available data, *M. zhewanensis* is confirmed from only two localities in Zhejiang and Anhui provinces, which likely represent two subpopulations. Preliminary estimates indicate a restricted Area of Occupancy (AOO) and a presumably small total population size. However, precise calculation of its Extent of Occurrence (EOO) and a comprehensive census of mature individuals are not yet available. Consequently, according to the IUCN Red List Categories and Criteria [40], this species is preliminarily assessed as Data Deficient (DD) due to the current uncertainties regarding its full distribution range and population parameters. Further field surveys are required to collect the necessary data for a definitive conservation status assessment.

Additional specimens examined (paratypes). CHINA. Zhejiang Province, Hangzhou City, Lin'an District, Qingliangfeng Town, Maxiao, Langchuan Village, Taizijian, in wet place of the valley, ~~alt.~~ 1,344 m ~~a.s.l.~~, 118°53′40.08"E, 30°10′29.16"N, Z. H. Chen LA24050402 (ZM); the same locality and date, Zhexitianlu, in damp place by small ditch edge, Z. H. Chen LA24050403 (ZM). Anhui Province, Xuancheng City, Jixi County, Fuling Town, Yonglai Village, on shaded and damp steep of forest margin of Carya cathayensis in valley, ~~alt.~~ 694 m ~~a.s.l.~~, 118°50′40.8"E, 30°8′52.56"N, 21 Jun. 2023, Z.H. Chen et H.W. Zhang

AH23062101, AH23062102 (ZM).

Key to the classification of pubescent-fruited closely related taxa of Mazus in China.

1. Plants without stolons, never producing adventitious roots at nodes.........................2

1. Plants with stolons, producing adventitious roots at nodes where touching ground..3

2. Stems erect; cauline leaves sessile; leaf blade oblong to oblanceolate, 2–7 cm long; pedicel shorter than or nearly equaling calyx; corolla deep bluish-purple..*M. stachydifolius*

2. Stems erect or ascending; cauline leaves with a winged petiole; leaf blade ovate-spatulate, 3.5–10 cm long; pedicel equal to or longer than calyx; corolla pale purple ....*M. caducifer*

3. Plants lacking a distinct erect stems; stolons slender and procumbent; leaf blade suborbicular or ovate, base truncate or broadly cuneate; petiole of cauline leaves 1.5–6.5 cm...4

3. Plants with both erect stems and stolons; stolons somewhat slender, arching, procumbent, or decumbent; leaf blade elliptic, base cuneate; petiole of cauline leaves 0.5–1.0 cm or inconspicuous... 5

4. Inflorescence terminal; upper lip of corolla with lobe apices irregularly dentate; calyx lobes nearly equal to tube; corolla 1.3–1.7 cm long..................*M. xiuningensis*

4. Inflorescence terminal and axillary; upper lip of corolla with lobe apices entire; calyx lobes longer than tube; corolla 0.9–1.1 cm long ....*M. jiangshiensi*

5. Stolons 10–30 cm long; leaves on erect stems obovate to obovate-spatulate, margin incised-serrate; corolla 0.8–1.2 cm long ....*M. spicatus*

5. Stolons 25–70(–100) cm long; leaves on erect stems elliptic, margin shallowly and densely serrate; corolla 1.4–2.0 cm long ....*M. zhewanensis*

Acknowledgements

We are deeply grateful to Mr. Jun Liu (Zhejiang University Library) for assistance in consulting the original literature, and to Professor Jian-wen Shao (Anhui Normal University) for providing high-resolution images of the type specimen of *M. xiuningensis*. We also grateful to Mr. Guo-fu Fang (Jixi Conservation Station of Anhui Qingliangfeng National Nature Reserve), Professor Yue-liang Xu (Zhejiang Natural History Museum), Professor Gen-you Li (Zhejiang A&F University), Professor Jin-bao Pu (Zhejiang Academy of Traditional Chinese Medicine), Senior Engineer Shang-jiao Ying, Senior Engineer Xi-yang Ye, Jian-ping Zhong, and Li-cheng Liang for their valuable support and collaboration during the field investigations. Special appreciations are extended to Ms. Yi Zhang for preparing the fine line drawings.

Additional information

Funding

This study was financially supported by Major Central Government Budget Increases and Decreases (2060302), the Project of Survey, File and Codification of Wild Plants and the Second Edition of Flora of Zhejiang (335010-2015-0005)，the Zhejiang Key Discipline in Traditional Chinese Medicine for Pharmaceutical Botany (2024-XK-06), the Investigation and Research on National Key Protected Wild Plants in Lin'an District (231012), and the Research on the Classification of Doubtful Plants in Zhejiang Province (ZFSPLD2025-01).

Ethical statement

No ethical statement was reported.

Author contributions

Conceptualization: Ke-Lang Lou, Zheng-Hai Chen; Methodology: Wei-Qing Liang, Zheng-Hai Chen; Investigation: Wei-Qing Liang, Wen-Yuan Xie, Zheng-Hai Chen, Jun-Ping Li, Hong-Wei Zhang, Pan Xu; Data curation: Ke-Lang Lou, Pan Xu; Software & visualization: Ke-Lang Lou, Feng Chen; Writing-original draft: Ke-Lang Lou, Wen-Yuan Xie; Writing-review & editing: Wei-Qing Liang, Zheng-Hai Chen. All authors approved the final manuscript.

Conflicts of Interest

The authors declare no conflict of interest.

Author ORCIDs

Ke-Lang Lou https://orcid.org/0009-0006-7095-9163

Wen-Yuan Xie https://orcid.org/0000-0003-1529-0787

Pan Xu https://orcid.org/0000-0002-3309-773X

Hong-Wei Zhang https://orcid.org/0009-0008-0264-0462

Feng Chen https://orcid.org/0000-0002-3560-6710

Jun-Ping Li https://orcid.org/0009-0000-7655-3849

Wei-Qing Liang https://orcid.org/0009-0003-2948-0845

Zheng-Hai Chen https://orcid.org/0000-0002-8852-7127

Data availability

All of the data that support the findings of this study are available in the main text.

References

[1] POWO (2025) Plants of the World Online. Facilitated by the Royal Botanic Gardens, Kew. https://powo.science.kew.org/ [Accessed 4 October 2025]

[2] Hong DY, Yang HB, Jin CL, Holmgren NH (1998) Scrophulariaceae. In: Wu ZY, Raven PH (Eds) Flora of China, Vol. 18. Science Press, Beijing & Missouri

Botanical Garden Press, St. Louis, 42–378.

[3] Fischer E (2004) Scrophulariaceae. In: Kadereit JW (Ed.) The Families and Genera of Vascular Plants, Vol. 7. Springer, Berlin, 333–432. https://doi.org/10.1007/978-3-642-18617-2_21

[4] Deng T, Zhang XS, Kim C, Zhang JW, Zhang DG, Volis S (2016) *Mazus sunhangii* (Mazaceae), a new species discovered in central China appears to be highly endangered. PLoS ONE 11(10): e0163581. https://doi.org/10.1371/journal.pone.0163581

[5] Lü ZY (2021) Taxonomic revision of Mazaceae worldwide. PhD Thesis: Yunnan Normal University, Kunming, 1–181.

[6] Yang HB (1979) Scrophulariaceae. In: Tsoong PC, Yang HB (Eds) Flora Reipublicae Popularis Sinicae, Vol. 67(2). Science Press, Beijing, 172–196.

[7] Qian XH (1991) Scrophulariaceae. In: Editorial Committee of Flora of Anhui (Ed.) Flora of Anhui, Vol. 4. Anhui Science and Technology Press, Hefei, 361–365. [In Chinese]

[8] Chen HB (1993) *Mazus*. In: Editorial Committee of Flora of Fujian (Ed.) Flora of Fujian, Vol. 5. Fujian Science and Technology Press, Fuzhou, 78–80. [In Chinese]

[9] Zheng CZ (1993) Flora of Zhejiang, Vol. 6. In: Editorial Committee of Flora of Zhejiang (Eds) Flora of Zhejiang. Zhejiang Science and Technology Press,

Hangzhou, 45–48. [In Chinese]

[10] Zheng CZ (2005) Manual for Identification of Seed Plants in Zhejiang. Zhejiang Science and Technology Press, Hangzhou, 337–338. [In Chinese]

[11] Qian SX (1999) Scrophulariaceae. In: Xu BS (Ed.) Flora of Shanghai, Vol. 1. Shanghai Scientific and Technological Literature Press, Shanghai, 569 – 570. [In Chinese]

[12] Liu RL, Zhang ZX, Liao WM (2010) Checklist of Seed Plants in Jiangxi. China Forestry Publishing House, Beijing, p. 260. [In Chinese]

[13] Song CF, Liu QX (2015) Scrophulariaceae. In: Liu QX (Ed.) Flora of Jiangsu, Vol. 4. Jiangsu Phoenix Science and Technology Press, Nanjing, 247–249. [In Chinese]

[14] Zhou Z (2021) *Mazus*. In: Ding BY (Ed.) Flora of Zhejiang (New Edition), Vol. 7. Zhejiang Science and Technology Press, Hangzhou, 437–442. [In Chinese]

[15] Guo XH, Liu XL (1990) A new species of *Mazus* from Anhui. Acta Phytotaxonmica Sinica 28(2): 163-164. [In Latin & Chinese]

[16] Maximowicz CJ (1875) Diagnoses des nouvelles plantes du Japon et de la Mandjourie. Bulletin de l'Académie Impériale des Sciences de Saint-Pétersbourg 20(3): 438–439.

[17] Hance HF (1882) Spicilegia florae sinensis: Diagnosis of new, and habitats of rare or hitherto unrecorded, Chinese plants. Journal of Botany, British and Foreign 20(238): 289–296.

[18] van Steenis CGGJ (1958) Miscellaneous botanical notes. Nova Guinea, New Series 9: 31.

[19] Makino T (1902) Observations on the Flora of Japan. Botanical Magazine 16(186): 153–162.

[20] Hemsley WB, Forbes FB (1890) An enumeration of all the plants known from China Proper, Formosa, Hainan, Corea, the Luchu Archipelago, and the Island of Hongkong, together with their distribution and synonymy. Journal of the Linnean Society, Botany 26(175): 121–236.

[21] Vaniot E (1905) Plantae Bodinierianae Scrophulariaceae. Bulletin de I'Académie Internationale de Géographie Botanique 15: 85–88.

[22] Tsoong PC (1954) Some new species of Scrophulariaceae from eastern Asia. Kew Bulletin 9(3): 445.

[23] Qian M, Geng LB (1987) A new species of *Mazus* from Fujian. Wuyi Science Journal 7: 263–265.

[24] Chen YB, Chen XY, Ma L, Zhao Z, Chen SP (2024) *Mazus jiangshiense* (Mazaceae), a new species from China: evidence from morphological and

molecular analyses. PhytoKeys 241: 81–90. https://doi.org/10.3897/phytokeys.241.117787

[25] Zhang XP, Guo XH, Xu JL (2000) New taxon of *Mazus* Lour (Scrophulariaceae) from Anhui. Journal of Anhui Normal University (Natural Science) 23(1): 33–35. [In Latin & Chinese]

[26] Zhang XW, Liu X, Xie WY , Mei XD, Wu DH, Chen F, Chen ZH (2025) A new species of Scrophulariaceae from eastern China: *Mazus jingtaiensis*. Journal of Hangzhou Normal University (Natural Science Edition) 24(4): 392–397. https://doi.org/10.19926/j.cnki.issn.1674-232X.2024.06.221

[27] Handel-Mazzetti H (1926) Plantae novae Sinenses, diagnosibus brevibus descriptae a Dre Henr. Anzeiger der Akademie der Wissenschaften in Wien, Mathematisch-Naturwissenschaftliche Klasse 43(3): 10.

[28] Jin XF, Xu SF, Xie JB, Gao J (2009) New materials of the seed plants in Zhejiang (III) . Journal of Zhejiang University (Science Edition) 36(5): 586–588.

[29] Olmstead RG, Michaels HJ, Scott KM, Palmer JD (1992) Monophyly of the Asteridae and identification of their major lineages inferred from DNA sequences of rbcL. Annals of the Missouri Botanical Garden 79: 249–265. https://doi.org/10.2307/2399768

[30] Bremer B, Bremer K, Heidari N, Erixon P, Olmstead RG, Anderberg AA,

Källersjö M, Barkhordarian E (2002) Phylogenetics of asterids based on 3 coding and 3 non-coding chloroplast DNA markers and the utility of non-coding DNA at higher taxonomic levels. Molecular Phylogenetics and Evolution 24:274–301. https://doi.org/10.1016/S1055-7903(02)00240-3

[31] Taberlet P, Gielly L, Pautou G, Bouvet J (1991) Universal primers for amplification of three non-coding regions of chloroplast DNA. Plant Molecular Biology 17(5): 1105–1109. https://doi.org/10.1007/BF00037152

[32] White TJ, Bruns TD, Lee SB, Taylor JW (1990) Amplification and direct sequencing of fungal ribosomal RNA genes for phylogenetics. In: Innis, MA, Gelfand DH, Sninsky JJ, White TJ (Eds.) PCR Protocols: A Guide to Methods and Applications. Academic Press, San Diego, 315–322. https://doi.org/10.1016/B978-0-12-372180-8.50042-1

[33] Linnaeus C (1753) Species Plantarum, Tomus. II. Holmiae [Stockholm]: Impensis Laurentii Salvii, 633.

[34] Hooker JD, Thomson T (1857) On three new Indian Scrophularineae. Hooker's Journal of Botany and Kew Gardens Miscellany 9: 244.

[35] Li B, Le XG, Min DZ, Xu L, Chen B (2022) *Mazus danxiacola* (Mazaceae), a distinct new species endemic to Danxia landform in Jiangxi Province, eastern China. PhytoKeys 199: 17–28. https://doi.org/10.3897/phytokeys.199.85717

[36] Wong TKF, Ly-Trong N, Ren H, Banos H, Roger AJ, Susko E, Bielow C, De Maio N, Goldman N, Hahn MW, Huttley G, Lanfear R, Minh BQ (2025) IQ-TREE 3: Phylogenomic Inference Software using Complex Evolutionary Models. Preprint by EcoEvoRxiv. https://doi.org/10.32942/X2P62N

[37] Kalyaanamoorthy S, Minh BQ, Wong TKF, Von Haeseler A, Jermiin LS (2017) ModelFinder: fast model selection for accurate phylogenetic estimates. Nature Methods 14: 587–589. https://doi.org/10.1038/nmeth.4285

[38] Hoang DT, Chernomor O, Von Haeseler A, Minh BQ, Vinh LS (2018) UFBoot2: Improving the Ultrafast Bootstrap Approximation. Molecular Biology and Evolution 35(2): 518–522. https://doi.org/10.1093/molbev/msx281

[39] Ronquist F, Teslenko M, Van der Mark P, Ayres DL, Darling A, Höhna S, Larget B, Liu L, Suchard MA, Huelsenbeck JP (2012) MrBayes 3.2: efficient Bayesian phylogenetic inference and model choice across a large model space. Systematic Biology 61(3): 539–542. https://doi.org/10.1093/sysbio/sys029

[40] IUCN (2025) The IUCN Red List of Threatened Species. Version 2025–2. https://www.iucnredlist.org [Accessed: 4 October 2025]

Click here to access/download;Figure;Fig 1.tif

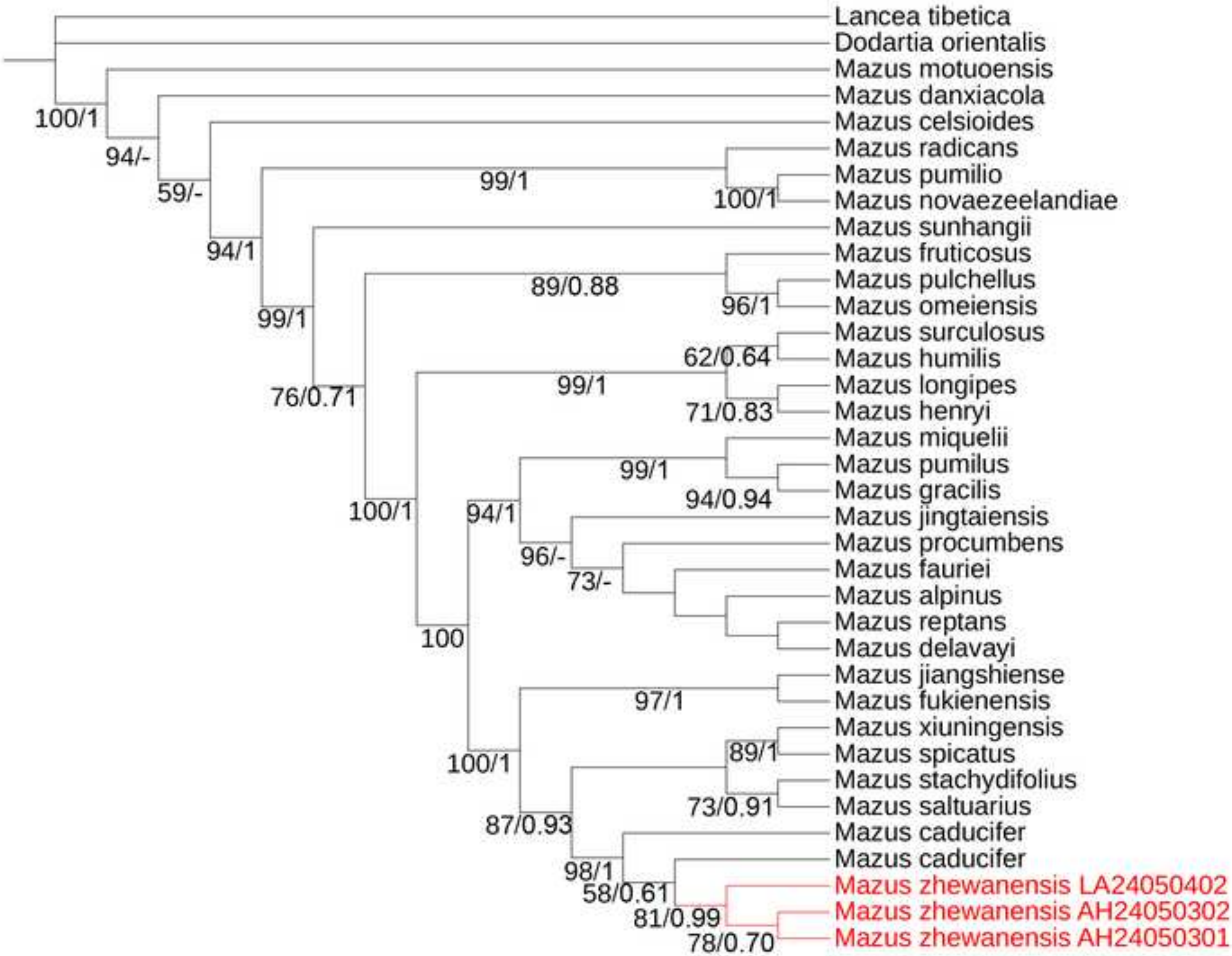

Click here to access/download;Figure;Fig 2.jpg

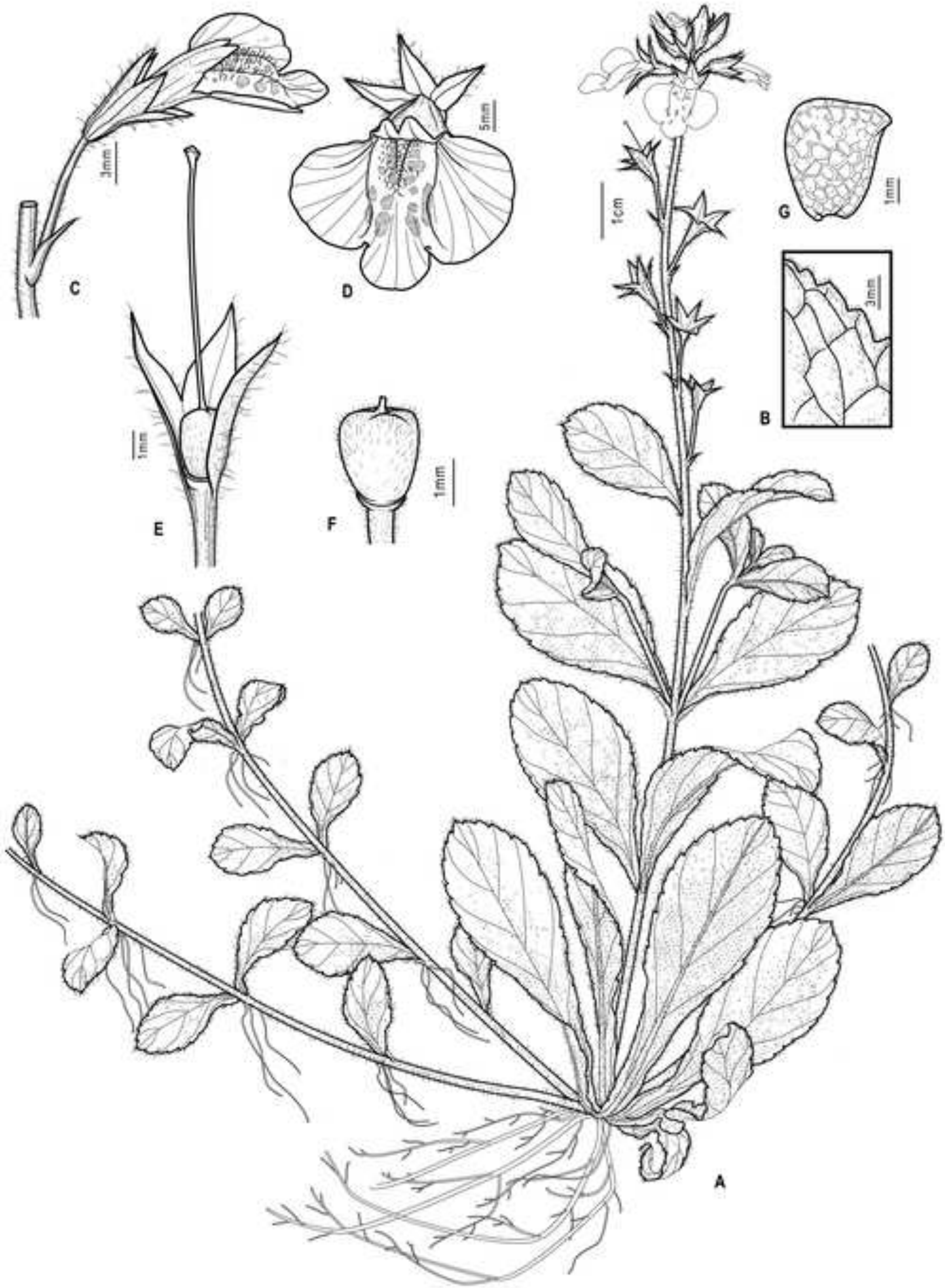

Click here to access/download;Figure;Fig 3.tif

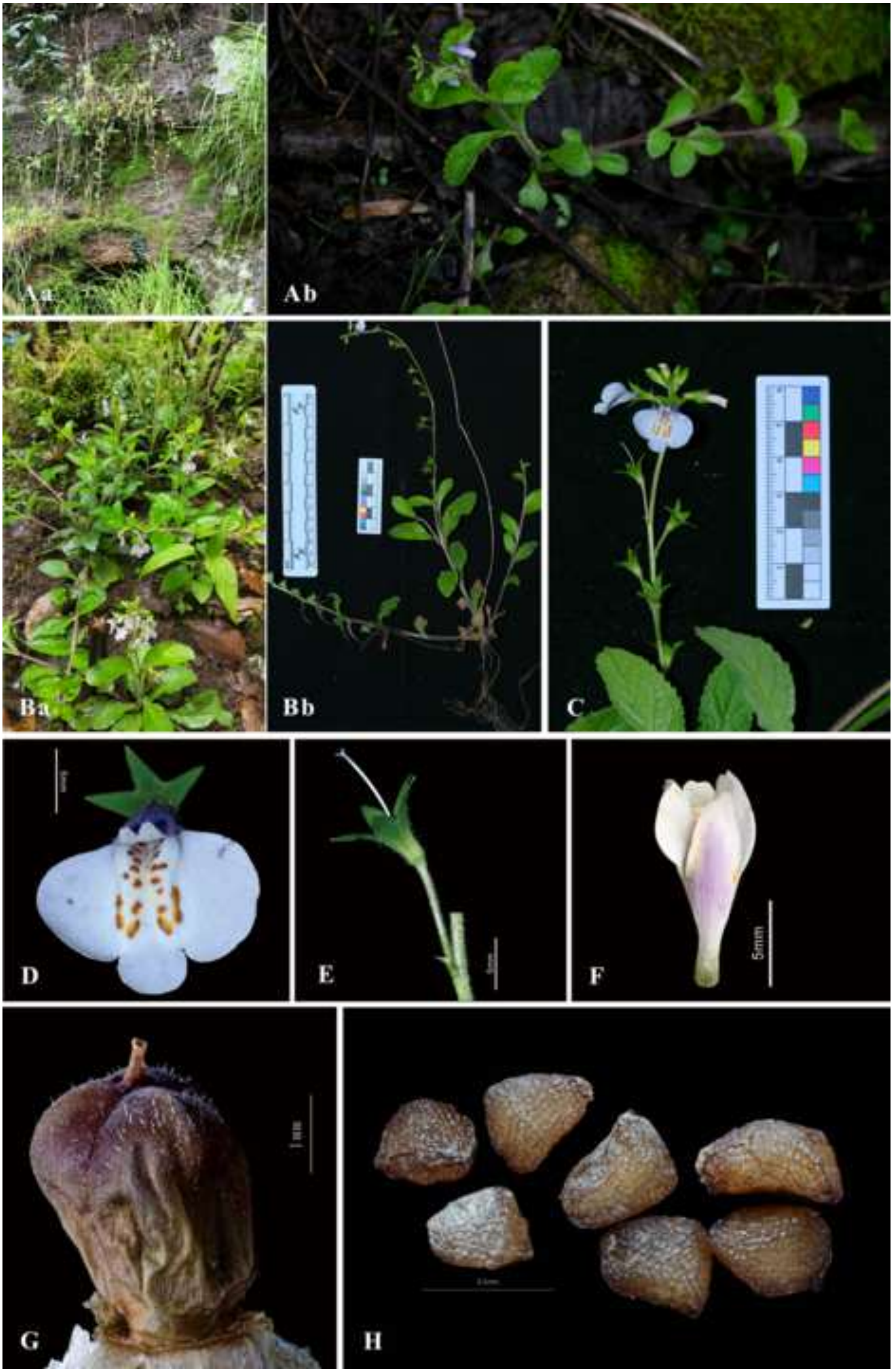

Click here to access/download;Figure;Fig 4.tif

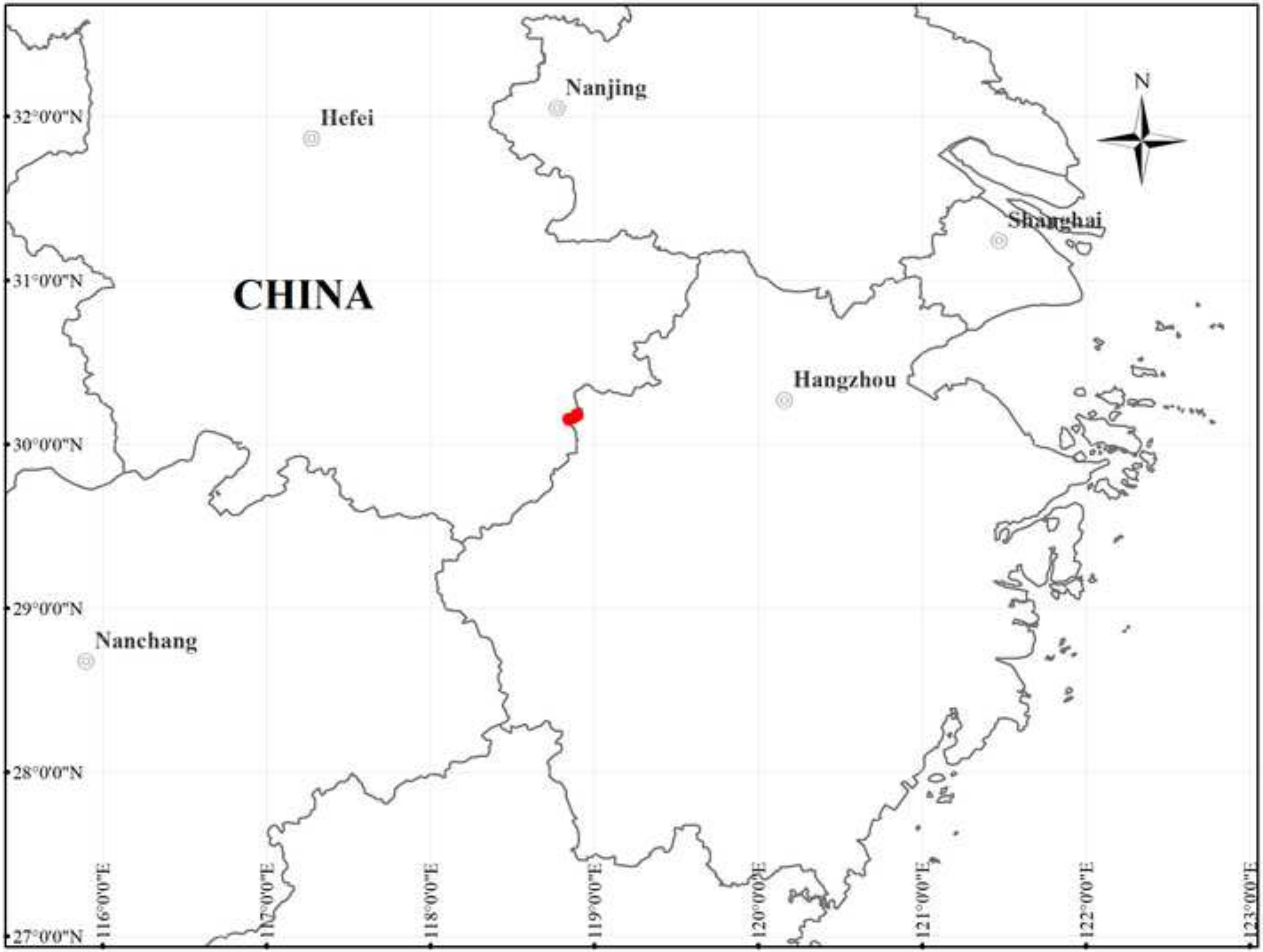

Supplement: Supplementary file 4 [file pone.0356991.s007.pdf]
