# Supplementary material for: Mazus zhewanensis (Mazaceae): A new species from Eastern China
Source: PLoS One. 2026 Oct 7;21(10):e0356991. doi: 10.1371/journal.pone.0356991 (PMC13645076; doi:10.1371/journal.pone.0356991)
Supplement: Supplementary file 5 [file pone.0356991.s008.docx]

**Response to Reviewers：**

We sincerely thank the editors and reviewers for the careful evaluation of our manuscript and for the constructive suggestions. These comments have been very helpful in improving the clarity, rigor, and practical relevance of the review. We have carefully revised the manuscript accordingly. Our point-by-point responses are provided below.

**Academic Editor**

**Comment 1:** Please ensure that your manuscript meets PLOS ONE's style requirements.

**Response:** We acknowledge the editor's concern regarding the manuscript formatting. We have revised the formatting throughout to comply with PLOS ONE's style requirements, including the title page, section headings, and file naming.

**Comment 2:** In your Methods section, please provide additional information regarding the permits you obtained for the work. Please ensure you have included the full name of the authority that approved the field site access and, if no permits were required, a brief statement explaining why.

**Response:** The collections were made outside protected areas and did not involve any endangered or protected species, and the study was conducted in compliance with all relevant local and national regulations.

**Comment 3:** 3. Thank you for submitting the above manuscript to PLOS ONE. During our internal evaluation of the manuscript, we found significant text overlap between your submission and previous work in the [introduction, conclusion, etc.].

We would like to make you aware that copying extracts from previous publications, especially outside the methods section, word-for-word is unacceptable. In addition, the reproduction of text from published reports has implications for the copyright that may apply to the publications.

Please revise the manuscript to rephrase the duplicated text, cite your sources, and provide details as to how the current manuscript advances on previous work. Please note that further consideration is dependent on the submission of a manuscript that addresses these concerns about the overlap in text with published work.

**Response:** We have substantially revised the Introduction and Conclusion sections to ensure all content is original. The revisions are visible in the marked-up manuscript, and we have added appropriate citations to clearly distinguish our work from previous studies.

**Comment 4:** Please state what role the funders took in the study. If the funders had no role, please state: "The funders had no role in study design, data collection and analysis, decision to publish, or preparation of the manuscript."

**Response:** We have revised the Role of Funder statement in the cover letter as requested, and it now reads: *"The funders had no role in study design, data collection and analysis, decision to publish, or preparation of the manuscript."*

**Comment 5:** Please note that supplementary tables (should remain/ be uploaded) as separate "supporting information" files.

**Response:** All supplementary tables have been uploaded as separate Supporting Information files via the submission system.

**Comment 6:** If the reviewer comments include a recommendation to cite specific previously published works, please review and evaluate these publications to determine whether they are relevant and should be cited. There is no requirement to cite these works unless the editor has indicated otherwise.

**Response:** We reviewed all reviewer comments and found no specific citation recommendations. Nevertheless, we have updated the Introduction and Discussion with relevant literature to provide broader context for our study.

**Comment 7:** 7. Please take this opportunity to be sure you have met all of our guidelines for new species. When publishing papers that describe a new botanical taxon, PLOS aims to comply with the requirements of the International Code of Nomenclature for algae, fungi, and plants (ICN). In association with the International Plant Names Index (IPNI), the following guidelines for publication in an online-only journal have been agreed such that any scientific botanical name published by us is considered effectively published under the rules of the Code. Please note that these guidelines differ from those for zoological nomenclature, and apply only to seed plants, ferns, and lycophytes.

Effective January 2012, "the description or diagnosis required for valid publication of the name of a new taxon" can be in either Latin or English. This does not affect the requirements for scientific names, which are still to be Latin.

Also effective January 2012, the electronic PDF represents a published work according to the ICN for algae, fungi, and plants. Therefore the new names contained in the electronic publication of a PLOS ONE article are effectively published under that Code from the electronic edition alone, so there is no longer any need to provide printed copies.

For proper registration of the new taxon, we require two specific statements to be included in your manuscript.

**Response:** In accordance with PLOS ONE's botanical guidelines, we have added a "Nomenclature" subsection to the Methods (Lines 145–157) and placed the placeholder "XXXXX" for the LSID under the new species name in the Results. We will obtain the LSID from IPNI upon acceptance.

We sincerely thank the Academic Editor for handling our manuscript and for the valuable comments that have substantially improved the work.

**Reviewer 1**

**Comment 1:** The phylogenetic analysis revealed that the newly described species is nested within the genetic clade of *M. caducifer*, suggesting a close affinity. To further clarify the taxonomic status and genetic divergence between these two closely related taxa, it is recommended that: Incorporate additional individual sequences of *M. caducifer* (newly generated or publicly available data from databases such as NCBI) into the dataset; Reconstruct the Neighbor-Joining (NJ) tree based on the expanded dataset to comprehensively compare the genetic differentiation and resolve the phylogenetic placement of the new species relative to *M. caducifer*.

**Response:** We thank the reviewer for this constructive suggestion. In response, we collected and sequenced additional specimens of *M. caducifer* from three geographically distinct localities. These newly obtained sequences were integrated with our previous dataset, and we reconstructed Neighbor-Joining (NJ), Maximum Likelihood (ML), and Bayesian Inference (BI) trees, along with genetic divergence and distance analyses. The updated phylogeny consistently resolves *M. zhewanensis* as a well-supported independent lineage and clearly clarifies its phylogenetic placement within the *M. caducifer* complex. These results provide further evidence supporting the distinctiveness of the new species.

**Revision location:** We have updated the Materials and Methods (Molecular section) and Results (Phylogenetic analyses) to reflect the expanded dataset and newly reconstructed trees, including the addition of a Neighbor-Joining (NJ) tree. Corresponding updates have also been made to Fig 2 and the relevant Supplementary Figures and Tables.

**Comment 2:** Significantly strengthen the comparative morphological analysis between the new species and *M. caducifer*.

**Response:** We fully agree with this suggestion. To address this, we conducted population-level morphological measurements (≥20 individuals per taxon) and systematically compared stable qualitative and quantitative characters between *M. zhewanensis* and *M. caducifer*. The morphological comparison between the two species has been substantially strengthened throughout the main text, Table 2, and the color plate, with stable diagnostic characters now clearly identified.

**Revision location:** We have revised the Results (Morphological comparison) section, updated Table 2, added a new color plate (Fig 1) incorporating *M. caducifer*, and expanded the Discussion accordingly.

We sincerely thank the reviewer again. These comments substantially improved the manuscript.

**Reviewer 2**

**Comment 1:** It would be nice to add *M. caducifer* in Table 2.

**Response:** Done. Table 2 has been expanded to include the new species, *M. caducifer*, *M. spicatus*, and other relevant taxa, with complete and directly comparable character matrices.

**Revision location:** Table 2 (replaced throughout the manuscript).

**Comment 2:** Table 2: The comparative characters should be described in a consistent format across all taxa.

**Response:** We have fully standardized the format throughout Table 2. Specifically, we have unified taxonomic terminology, units of measurement, and the expression of numerical ranges across all taxa. In addition, we have clearly indicated sample sizes, measurement protocols, and data sources (own measurements vs. literature-derived data) in the table footnote and corresponding Materials and Methods section.

**Revision location:** We have revised the Table 2 footnote and accompanying notes, and updated the Materials and Methods (Morphometric measurements) section accordingly.

**Comment 3:** Figure 1. Two samples of *M. caducifer* do not form a monophyletic. It seems that *M. zhewanensis* is not sister to *M. caducifer*, but it merge in *M. caducifer* clade. Could the authors provide more information?

**Response:** We thank the reviewer for this careful observation. In response, we expanded our sampling of *M. caducifer* to five accessions and reanalyzed the data. The updated phylogeny shows that *M. zhewanensis* is nested within the *M. caducifer* complex but forms a distinct, well-supported lineage. Separate analyses of the ITS and chloroplast datasets revealed cytonuclear discordance, with the ITS data alone showing weak resolution within this group. In the revised Discussion, we have addressed the potential effects of marker choice and cytonuclear discordance on the recovery of monophyly. Despite this, the chloroplast dataset consistently supports *M. zhewanensis* as an independent lineage, corroborated by morphological evidence.

**Revision location:** We have revised the Results (Phylogenetic analyses) section, updated Fig 2 with the expanded dataset, added Supplementary Figures S1–S3 (ITS separate trees), and expanded the Discussion to address cytonuclear discordance and its implications.

We sincerely thank the reviewer again. These comments substantially improved the manuscript.

**Reviewer 3**

**Comment 1:** The main differences between the proposed new species and the morphologically similar species *M. spicatus* are largely based on quantitative characters. I consider these characters to be unreliable, as they likely represent intraspecific variation rather than true diagnostic traits. In contrast, this taxon appears to be more similar to *M. caducifer*, and it is more plausible that it represents ecological variation of *M. caducifer* under specific habitat conditions. Therefore, the recognition of this taxon as a new species is not justified.

**Response:** We appreciate the reviewer’s critical assessment. We agree that quantitative characters alone are insufficient for species delimitation. In response, we have: (1) prioritized morphological comparison with *M. caducifer* over *M. spicatus*; (2) conducted population-level sampling and measurements (≥20 living plants of the new species) to distinguish stable qualitative traits from quantitative characters with overlapping ranges; (3) provided multiple independent lines of evidence (genetic distances, phylogenetic signals, and morphological data) to rule out ecological plasticity; and (4) explicitly listed stable, non-overlapping morphological diagnostic characters and molecular diagnostic sites in the main text. Together, these lines of evidence support the recognition of *M. zhewanensis* as an independent lineage. Critically, although the chloroplast and ITS datasets show different levels of resolution, all available evidence consistently indicates that *M. zhewanensis* represents an independently evolving lineage with stable morphological diagnostic characters.

**Revision location:** We have revised the Introduction, Materials and Methods, Results, and Discussion sections, as well as Table 2, to incorporate these changes.

**Comment 2:** The manuscript has not been carefully prepared. For example, the introduction is overly simplistic. As a taxonomic paper, it should provide essential background information on the genus, such as: its taxonomic history; the number of species in China and how many are endemic; recent advances in molecular phylogenetic studies; the main diagnostic characters of the genus; and the criteria used for species delimitation. In addition, *Mazus* exhibits substantial morphological variation. The authors should conduct more comprehensive population-level morphological studies, focusing on a broader sampling of species, rather than treating species delimitation as a superficial “spot-the-difference” exercise to justify the publication of new taxa.

**Response:** We acknowledge that the Introduction was insufficient and have accordingly rewritten and substantially expanded it. The revised Introduction now includes: (1) the taxonomic history and contentious issues surrounding the *Mazus*; (2) a summary of species diversity in China, including total species richness (ca. 30 species); (3) recent advances in molecular phylogenetic studies of *Mazus*, with particular attention to species complexes; (4) the core diagnostic characters of the genus; and (5) the integrative species delimitation criteria adopted in this study (combining morphological, molecular, and geographic/ecological evidence). In addition, we explicitly clarify that our study is based on population-level morphological and molecular sampling, rather than relying solely on a few diagnostic differences.

**Revision location:** We have thoroughly revised the Introduction section and updated the Materials and Methods to clarify the population-level sampling approach.

**Comment 3:** Please provide complete and detailed collection information, including herbarium barcode numbers. In the Materials and Methods section, the authors should specify which type specimens were examined and how many specimens across the genus were studied. These data should also be summarized in a supplementary table.

**Response:** We thank the reviewer for this suggestion. In the revised manuscript, we have provided detailed collection information for all examined specimens. A total of over 120 specimens representing more than 20 species of *Mazus* were examined, deposited in PE, ZM, ZJMR, IBSC, NAS, HZ, ANUB, FJFC, CSH, K, E, WU, TAI, GH, NY, PH, US, KYO, HNWP, WUK, A, and BM. These include type specimens of *M. spicatus* (holotype E00284108, syntype E00284109), *M. caducifer* (holotype BM), *M. xiuningensis* (holotype ANUB13023341, paratype ANUB13023647), *M. jiangshiense* (holotype FJFC592371, isotype CSH592372), *M. stachydifolius* (isotype TAI119106), *M. saltuarius* (holotype WU0060245, isotypes E00284120, K001079339), and numerous other types. Complete specimen information, including herbarium codes and barcode numbers, is summarized in Table S1 (Supporting Information).

**Revision location:** We have revised the Materials and Methods (Taxon sampling) section and added Table S1 (list of examined specimens) as Supporting Information.

**Comment 4:** L109–110: Which characters mentioned above were used to distinguish *M. spicatus* from the "new species"?

**Response:** We thank the reviewer for this question. In the revised Morphological comparison section, we have explicitly listed the diagnostic characters distinguishing *M. zhewanensis* from its close relatives. From *M. spicatus*, it differs in leaf shape (elliptic with shallowly and densely serrate margins vs. obovate to obovate-spatulate with incised-serrate margins) and corolla size (1.4–2.0 cm vs. 0.8–1.2 cm). From *M. caducifer*, it differs in stolon rooting condition (rooting at nodes vs. no rooting) and calyx shape (campanulate vs. funnelform). These diagnostic traits have been highlighted in bold in Table 2 for easy reference.

**Revision location:** We have revised the Morphological comparison section and updated Table 2 with diagnostic characters highlighted in bold for easy reference.

**Comment 5:** In Table 2, four taxa are listed. I suggest preparing a color figure showing these species to facilitate morphological comparison. The authors mention that some characters are based on “own measurements”—how many specimens (or individuals) were measured, and which characters were included?

**Response:** We have fully addressed these concerns. Specifically: (1) in the Materials and Methods, we now specify that morphological measurements of *M. zhewanensis* were taken from ≥20 living plants in the field, with voucher specimens deposited at ZJMR, ZM, and PE; (2) we have removed the ambiguous phrase "own measurements" and replaced it with "field measurements of living plants (≥20 individuals)" for the new species, with data for other taxa derived from literature and examination of herbarium specimens; (3) we have added a new color comparative plate (Fig 1) illustrating the new species together with *M. caducifer* and *M. spicatus*; and (4) we have annotated Table 2 with sample sizes and data sources in the footnote.

**Revision location:** We have revised the Materials and Methods (Morphometric measurements) section, updated the Table 2 footnote, and added a new color plate as Fig 1.

**Comment 6:** What are the diagnostic features of the DNA sequences? These are not described.

**Response:** We thank the reviewer for pointing out this omission. In the revised manuscript, we have added a new subsection “Molecular diagnostic characters” in the Results section. It lists fixed nucleotide substitutions in the chloroplast regions (*rps16*, *rbcL*, and *trnL-trnF*) that distinguish *M. zhewanensis* from its closest relative, *M. caducifer*, with alignment positions and nucleotide states clearly indicated. These diagnostic sites are summarized in Table 3. In addition, the Kimura 2-parameter (K2P) genetic distance between the two species, calculated using the ape package in R, is also reported to further support their genetic distinctiveness.

**Revision location:** We have updated the Results (Molecular diagnostic characters) section and added Table S3 as Supporting Information.

**Comment 7:** Line 119: A bootstrap support (BP) of 81% and posterior probability (PP) of 0.99 do not constitute strong support. In the phylogenetic tree, the putative “new species” clusters with *M. caducifer*. Why did the authors not compare it directly with this species? In my opinion, they likely represent the same species, and the proposed new species is therefore invalid.

**Response:** We thank the reviewer for raising this concern. We have addressed this issue substantively in three ways. (1) With expanded sampling, the chloroplast phylogeny now supports the monophyly of *M. zhewanensis* with ML = 95% and BI = 1, and its sister relationship to *M. caducifer* with ML = 96% and BI = 1, providing stronger evidence than the original analysis. (2) Throughout the revised manuscript, we have made *M. caducifer* the primary comparison, integrating morphological, molecular, and ecological evidence to support the recognition of *M. zhewanensis* as an independent lineage. (3) In the Discussion, we have explained the initially modest support as due to limited phylogenetic signal in the ITS region, and we clarify that the chloroplast dataset now provides robust support for the distinctiveness of the new species.

**Revision location:** We have revised the relevant text in the Results (Phylogenetic analyses) and Discussion sections, and replaced Fig 2 with the updated phylogeny based on expanded sampling.

**Comment 8:** At present, too many so-called “new species” are being published (not limited to this genus), which creates confusion in taxonomy. Moreover, the authors of this study do not appear to have conducted in-depth taxonomic research on this genus. Some of the co-authors are affiliated with hospitals, which, although beyond the strict scope of this review, raises concerns about the level of taxonomic expertise. This is also reflected in certain unprofessional aspects of the manuscript. For instance, one of the naming authors of the “new species,” J.B. Pu, is not listed as an author of the paper—how can they be designated as a naming authority?

**Response:** We thank the reviewer for these critical comments and the opportunity to clarify our team's qualifications and taxonomic rigor.

**First, regarding J.B. Pu's authorship:** We fully agree with the reviewer and have corrected this oversight. Jin-bao Pu is now included as a full co-author in the revised manuscript, with his contributions to field collection, morphological identification, and nomenclature properly recognized in the author list and Author Contributions section.

**Second, regarding institutional affiliations:** We appreciate this concern and clarify that our team is jointly affiliated with the **Zhejiang Academy of Traditional Chinese Medicine** and **Tongde Hospital of Zhejiang Province**, which operate under a single administrative system. The Academy is an established research institute with long‑term expertise in medicinal plant taxonomy and herbarium‑based studies.

**Third, regarding taxonomic rigor:** To address concerns about poorly justified new species, our conclusions are based on integrative evidence—morphological (field measurements of ≥20 living plants of the new species, supplemented by herbarium specimens), molecular (expanded phylogenetic analyses with separate ITS and cpDNA trees, diagnostic nucleotide sites), and geographic/ecological data—with complete specimen information (barcode numbers) and type specimen photographs provided for full reproducibility.

**Revision location:** We have updated the authorship page to include J.B. Pu as a co-author, revised the Author Contributions and removed his name from the Acknowledgements, updated institutional affiliations, and expanded the Discussion to clarify our integrative taxonomic standards.

**Comment 9:** Please provide the barcode number and photographs of the type specimen.

**Response:** We thank the reviewer for this suggestion. In the Taxonomic treatment section, we have listed the barcode numbers and herbarium deposit locations for the holotype and isotype specimens in the type citation. A complete list of all examined specimens, including their barcodes, is provided in Table S1 (Supporting Information). In addition, a photograph of the holotype has been included as Fig 5 in the main text.

**Revision location:** We have updated the Taxonomic treatment section with barcode numbers in the type citation, added Table S1 (complete list of examined specimens) as Supporting Information, and included Fig 5 (photograph of the holotype) in the main text.

**Comment 10:** The identification key is also problematic. Some characters are not suitable for distinguishing species—for example, how can one reliably separate ranges such as 2–7 cm versus 3.5–10 cm?

**Response:** We thank the reviewer for this critical comment. We have completely revised the identification key. We minimized the use of overlapping quantitative characters and prioritized stable qualitative characters wherever possible. At critical nodes, we have avoided reliance on continuous measurements. The revised key is now based primarily on discrete, reliably observable characters.

**Revision location:** We have replaced the identification key throughout the manuscript.

**Comment 11:** Please provide phylogenetic trees based on both ITS and cpDNA datasets.

**Response:** We thank the reviewer for this suggestion. We have constructed separate phylogenetic trees for the ITS and chloroplast datasets using NJ, ML, and BI methods. A partition homogeneity test (ILD) revealed significant incongruence between the two datasets, indicating conflicting phylogenetic signals; a combined analysis was therefore not performed. The ITS trees showed weak resolution within the *M. caducifer* complex, with bootstrap support generally below 50% and individuals of *M. zhewanensis* and *M. caducifer* intermingled (Supplementary Figs S1–S3). In contrast, the chloroplast trees recovered *M. zhewanensis* as a well-supported monophyletic clade (Fig 2). The three ITS trees (NJ, ML, BI) are presented in Supplementary Figures S1–S3.

**Revision location:** We have updated the Materials and Methods and Results sections, updated Fig 2, and added Supplementary Figures S1–S3 (ITS NJ, ML, and BI trees) accordingly.

We sincerely thank the reviewer again. These comments substantially improved the manuscript.
