# Supplementary material for: Mazus zhewanensis (Mazaceae): A new species from Eastern China
Source: PLoS One. 2026 Oct 7;21(10):e0356991. doi: 10.1371/journal.pone.0356991 (PMC13645076; doi:10.1371/journal.pone.0356991)
Supplement: Peer review history

# Peer review: Decision Letter 0

**DOI:** 10.1371/journal.pone.0356991.r001

**Authors:** Renwu Wu

28 Apr 2026

PONE-D-26-09521

Mazus zhewanensis  (Mazaceae), a new species from Eastern China

PLOS One

Dear Dr. Lou,

Thank you for submitting your manuscript to PLOS ONE. After careful consideration, we feel that it has merit but does not fully meet PLOS ONE’s publication criteria as it currently stands. Therefore, we invite you to submit a revised version of the manuscript that addresses the points raised during the review process.

Please submit your revised manuscript by Jun 12 2026 11:59PM. If you will need more time than this to complete your revisions, please reply to this message or contact the journal office at plosone@plos.org. When you're ready to submit your revision, log on to https://www.editorialmanager.com/pone/ and select the 'Submissions Needing Revision' folder to locate your manuscript file.

Please include the following items when submitting your revised manuscript:

- A letter that responds to each point raised by the academic editor and reviewer(s). You should upload this letter as a separate file labeled 'Response to Reviewers'.
- A marked-up copy of your manuscript that highlights changes made to the original version. You should upload this as a separate file labeled 'Revised Manuscript with Track Changes'.
- An unmarked version of your revised paper without tracked changes. You should upload this as a separate file labeled 'Manuscript'.

If you would like to make changes to your financial disclosure, please include your updated statement in your cover letter. Guidelines for resubmitting your figure files are available below the reviewer comments at the end of this letter.

If applicable, we recommend that you deposit your laboratory protocols in protocols.io to enhance the reproducibility of your results. Protocols.io assigns your protocol its own identifier (DOI) so that it can be cited independently in the future. For instructions see: https://journals.plos.org/plosone/s/submission-guidelines#loc-laboratory-protocols. Additionally, PLOS ONE offers an option for publishing peer-reviewed Lab Protocol articles, which describe protocols hosted on protocols.io. Read more information on sharing protocols at https://plos.org/protocols?utm_medium=editorial-email&utm_source=authorletters&utm_campaign=protocols.

As the corresponding author, your ORCID iD is verified in the submission system and will appear in the published article. PLOS supports the use of ORCID, and we encourage all coauthors to register for an ORCID iD and use it as well. Please encourage your coauthors to verify their ORCID iD within the submission system before final acceptance, as unverified ORCID iDs will not appear in the published article. Only the individual author can complete the verification step; PLOS staff cannot verify ORCID iDs on behalf of authors.

We look forward to receiving your revised manuscript.

Kind regards,

Renwu Wu

Academic Editor

PLOS One

Journal Requirements:

When submitting your revision, we need you to address these additional requirements.

1. Please ensure that your manuscript meets PLOS ONE's style requirements, including those for file naming. The PLOS ONE style templates can be found at

https://journals.plos.org/plosone/s/file?id=wjVg/PLOSOne_formatting_sample_main_body.pdf and

https://journals.plos.org/plosone/s/file?id=ba62/PLOSOne_formatting_sample_title_authors_affiliations.pdf

2. In your Methods section, please provide additional information regarding the permits you obtained for the work. Please ensure you have included the full name of the authority that approved the field site access and, if no permits were required, a brief statement explaining why.

3. Thank you for submitting the above manuscript to PLOS ONE. During our internal evaluation of the manuscript, we found significant text overlap between your submission and previous work in the [introduction, conclusion, etc.].

We would like to make you aware that copying extracts from previous publications, especially outside the methods section, word-for-word is unacceptable. In addition, the reproduction of text from published reports has implications for the copyright that may apply to the publications.

Please revise the manuscript to rephrase the duplicated text, cite your sources, and provide details as to how the current manuscript advances on previous work. Please note that further consideration is dependent on the submission of a manuscript that addresses these concerns about the overlap in text with published work.

[If the overlap is with the authors’ own works: Moreover, upon submission, authors must confirm that the manuscript, or any related manuscript, is not currently under consideration or accepted elsewhere. If related work has been submitted to PLOS ONE or elsewhere, authors must include a copy with the submitted article. Reviewers will be asked to comment on the overlap between related submissions (http://journals.plos.org/plosone/s/submission-guidelines#loc-related-manuscripts).]

We will carefully review your manuscript upon resubmission and further consideration of the manuscript is dependent on the text overlap being addressed in full. Please ensure that your revision is thorough as failure to address the concerns to our satisfaction may result in your submission not being considered further

4. Thank you for stating the following financial disclosure:

"This study was financially supported by Major Central Government Budget Increases and Decreases (2060302), the Project of Survey, File and Codification of Wild Plants and the Second Edition of Flora of Zhejiang (335010-2015-0005)， the Zhejiang Key Discipline in Traditional Chinese Medicine for Pharmaceutical Botany (2024-XK-06), the Investigation and Research on National Key Protected Wild Plants in Lin'an District (231012), and the Research on the Classification of Doubtful Plants in Zhejiang Province (ZFSPLD2025-01)."

Please state what role the funders took in the study.  If the funders had no role, please state: "The funders had no role in study design, data collection and analysis, decision to publish, or preparation of the manuscript."

If this statement is not correct you must amend it as needed.

Please include this amended Role of Funder statement in your cover letter; we will change the online submission form on your behalf.

5. Please note that supplementary tables (should remain/ be uploaded) as separate "supporting information" files.

6. If the reviewer comments include a recommendation to cite specific previously published works, please review and evaluate these publications to determine whether they are relevant and should be cited. There is no requirement to cite these works unless the editor has indicated otherwise.

7. Please take this opportunity to be sure you have met all of our guidelines for new species. When publishing papers that describe a new botanical taxon, PLOS aims to comply with the requirements of the International Code of Nomenclature for algae, fungi, and plants (ICN). In association with the International Plant Names Index (IPNI), the following guidelines for publication in an online-only journal have been agreed such that any scientific botanical name published by us is considered effectively published under the rules of the Code. Please note that these guidelines differ from those for zoological nomenclature, and apply only to seed plants, ferns, and lycophytes.

Effective January 2012, "the description or diagnosis required for valid publication of the name of a new taxon" can be in either Latin or English. This does not affect the requirements for scientific names, which are still to be Latin.

Also effective January 2012, the electronic PDF represents a published work according to the ICN for algae, fungi, and plants. Therefore the new names contained in the electronic publication of a PLOS ONE article are effectively published under that Code from the electronic edition alone, so there is no longer any need to provide printed copies.

For proper registration of the new taxon, we require two specific statements to be included in your manuscript.

a.        In the Methods section, include a sub-section called "Nomenclature" using the following wording:

The electronic version of this article in Portable Document Format (PDF) in a work with an ISSN or ISBN will represent a published work according to the International Code of Nomenclature for algae, fungi, and plants, and hence the new names contained in the electronic publication of a PLOS ONE article are effectively published under that Code from the electronic edition alone, so there is no longer any need to provide printed copies.

In addition, new names contained in this work have been submitted to IPNI, from where they will be made available to the Global Names Index. The IPNI LSIDs can be resolved and the associated information viewed through any standard web browser by appending the LSID contained in this publication to the prefix http://ipni.org/. The online version of this work is archived and available from the following digital repositories: [INSERT NAMES OF DIGITAL REPOSITORIES WHERE ACCEPTED MANUSCRIPT WILL BE SUBMITTED (PubMed Central, LOCKSS etc)].

All PLOS ONE articles are deposited in PubMed Central and LOCKSS. If your institute, or those of your co-authors, has its own repository, we recommend that you also deposit the published online article there and include the name in your article.

b.   In the Results section, the globally unique identifier (GUID), currently in the form of a Life Science Identifier (LSID), should be listed under the new species name, for example:

Solanum aspersum S.Knapp, sp. nov. [urn:lsid:ipni.org:names:77103633-1] Type: Colombia. Putumayo: vertiente oriental de la Cordillera, entre Sachamates y San Francisco de Sibundoy, 1600-1750 m, 30 Dec 1940, J. Cuatrecasas 11471 (holotype, COL; isotypes, F [F-1335119], US [US-1799731]).

PLOS ONE staff will follow up with you after your manuscript is accepted for publication to provide instructions on contacting IPNI to obtain the GUID (LSID), and this information will then be added to the manuscript at that time. You may indicate where the number or numbers will be added using XXXXX or an equivalent placeholder.

A complete explanation of our guidelines for publishing new species can be found on our website: http://www.plosone.org/static/guidelines#botanical

Reviewers' comments:

Reviewer's Responses to Questions

**Comments to the Author**

1. Is the manuscript technically sound, and do the data support the conclusions?

Reviewer #1: Partly

Reviewer #2: Yes

Reviewer #3: No

**********

2. Has the statistical analysis been performed appropriately and rigorously?

Reviewer #1: Yes

Reviewer #2: N/A

Reviewer #3: No

**********

3. Have the authors made all data underlying the findings in their manuscript fully available?

The PLOS Data policy

Reviewer #1: Yes

Reviewer #2: Yes

Reviewer #3: Yes

**********

4. Is the manuscript presented in an intelligible fashion and written in standard English?

Reviewer #1: Yes

Reviewer #2: Yes

Reviewer #3: No

**********

Reviewer #1: The paper describes a new species of Mazus,and the manuscript is acceptable for publication after revision.

Revision suggestions:

1.The phylogenetic analysis revealed that the newly described species is nested within the genetic clade of Mazus caducifer, suggesting a close affinity. To further clarify the taxonomic status and genetic divergence between these two closely related taxa, it is recommended that: Incorporate additional individual sequences of M. caducifer (newly generated or publicly available data from databases such as NCBI) into the dataset; Reconstruct the Neighbor-Joining (NJ) tree based on the expanded dataset to comprehensively compare the genetic differentiation and resolve the phylogenetic placement of the new species relative to M. caducifer.

2.Significantly strengthen the comparative morphological analysis between the new species and Mazus caducifer.

Reviewer #2: The authors describe a new species of the genus Mazus from East China, based on morphological and molecular data. I have a few comments below:

1. It would be nice to add M. caducifer in Table 2.

2. Table 2: The comparative characters should be described in a consistent format across all taxa.

3. Figure 1. Two samples of M. caducifer do not form a monophyletic. It seems that M. zhewanensis is not sister to M. caducifer, but it merge in M. caducifer clade. Could the authors provide more information?

Reviewer #3: The main differences between the proposed new species and the morphologically similar species M. spicatus are largely based on quantitative characters. I consider these characters to be unreliable, as they likely represent intraspecific variation rather than true diagnostic traits. In contrast, this taxon appears to be more similar to M. caducifer, and it is more plausible that it represents ecological variation of M. caducifer under specific habitat conditions. Therefore, the recognition of this taxon as a new species is not justified.

The manuscript has not been carefully prepared. For example, the introduction is overly simplistic. As a taxonomic paper, it should provide essential background information on the genus, such as: its taxonomic history; the number of species in China and how many are endemic; recent advances in molecular phylogenetic studies; the main diagnostic characters of the genus; and the criteria used for species delimitation. In addition, Mazus exhibits substantial morphological variation. The authors should conduct more comprehensive population-level morphological studies, focusing on a broader sampling of species, rather than treating species delimitation as a superficial “spot-the-difference” exercise to justify the publication of new taxa.

Taxon sampling: Please provide complete and detailed collection information, including herbarium barcode numbers. In the Materials and Methods section, the authors should specify which type specimens were examined and how many specimens across the genus were studied. These data should also be summarized in a supplementary table.

L109–110: Which characters mentioned above were used to distinguish M. spicatus from the “new species”?

In Table 2, four taxa are listed. I suggest preparing a color figure showing these species to facilitate morphological comparison. The authors mention that some characters are based on “own measurements”—how many specimens (or individuals) were measured, and which characters were included?

What are the diagnostic features of the DNA sequences? These are not described.

Line 119: A bootstrap support (BP) of 81% and posterior probability (PP) of 0.99 do not constitute strong support. In the phylogenetic tree, the putative “new species” clusters with M. caducifer. Why did the authors not compare it directly with this species? In my opinion, they likely represent the same species, and the proposed new species is therefore invalid.

At present, too many so-called “new species” are being published (not limited to this genus), which creates confusion in taxonomy. Moreover, the authors of this study do not appear to have conducted in-depth taxonomic research on this genus. Some of the co-authors are affiliated with hospitals, which, although beyond the strict scope of this review, raises concerns about the level of taxonomic expertise. This is also reflected in certain unprofessional aspects of the manuscript. For instance, one of the naming authors of the “new species,” J.B. Pu, is not listed as an author of the paper—how can they be designated as a naming authority?

Please provide the barcode number and photographs of the type specimen.

The identification key is also problematic. Some characters are not suitable for distinguishing species—for example, how can one reliably separate ranges such as 2–7 cm versus 3.5–10 cm?

Please provide phylogenetic trees based on both ITS and cpDNA datasets. Previous studies have shown clear cytonuclear conflict in this group, yet the authors have not conducted separate analyses.

**********

what does this mean?). If published, this will include your full peer review and any attached files.

If you choose “no”, your identity will remain anonymous but your review may still be made public.

**Do you want your identity to be public for this peer review?**  For information about this choice, including consent withdrawal, please see our Privacy Policy

Reviewer #1: No

Reviewer #2: No

Reviewer #3: No

**********

[NOTE: If reviewer comments were submitted as an attachment file, they will be attached to this email and accessible via the submission site. Please log into your account, locate the manuscript record, and check for the action link "View Attachments". If this link does not appear, there are no attachment files.]

To ensure your figures meet our technical requirements, please review our figure guidelines: https://journals.plos.org/plosone/s/figures

You may also use PLOS’s free figure tool, NAAS, to help you prepare publication quality figures: https://journals.plos.org/plosone/s/figures#loc-tools-for-figure-preparation.

NAAS will assess whether your figures meet our technical requirements by comparing each figure against our figure specifications.

Attachment Submitted filename: PONE-D-26-09521.pdf

# Author comment: Author response to Decision Letter 1

**DOI:** 10.1371/journal.pone.0356991.r002

23 Jul 2026

Our detailed responses to the reviewers are provided in the uploaded file.

Attachment Submitted filename: Response to Reviewers.docx

# Peer review: Decision Letter 1

**DOI:** 10.1371/journal.pone.0356991.r003

**Authors:** Renwu Wu

11 Aug 2026

Mazus zhewanensis  (Mazaceae), a new species from Eastern China

PONE-D-26-09521R1

Dear Dr. Lou,

We’re pleased to inform you that your manuscript has been judged scientifically suitable for publication and will be formally accepted for publication once it meets all outstanding technical requirements.

Within one week, you’ll receive an e-mail detailing the required amendments. When these have been addressed, you’ll receive a formal acceptance letter and your manuscript will be scheduled for publication.

An invoice will be generated when your article is formally accepted. Please note, if your institution has a publishing partnership with PLOS and your article meets the relevant criteria, all or part of your publication costs will be covered. Please make sure your user information is up-to-date by logging into Editorial Manager at Editorial Manager® and clicking the ‘Update My Information' link at the top of the page. For questions related to billing, please contact billing support.

If your institution or institutions have a press office, please notify them about your upcoming paper to help maximize its impact. If they’ll be preparing press materials, please inform our press team as soon as possible -- no later than 48 hours after receiving the formal acceptance. Your manuscript will remain under strict press embargo until 2 pm Eastern Time on the date of publication. For more information, please contact onepress@plos.org.

Kind regards,

Renwu Wu

Academic Editor

PLOS One

Reviewers' comments:

Reviewer's Responses to Questions

**Comments to the Author**

Reviewer #1: All comments have been addressed

Reviewer #2: All comments have been addressed

**********

2. Is the manuscript technically sound, and do the data support the conclusions?

Reviewer #1: Yes

Reviewer #2: Yes

**********

3. Has the statistical analysis been performed appropriately and rigorously?

Reviewer #1: Yes

Reviewer #2: (No Response)

**********

4. Have the authors made all data underlying the findings in their manuscript fully available?

The PLOS Data policy

Reviewer #1: Yes

Reviewer #2: (No Response)

**********

5. Is the manuscript presented in an intelligible fashion and written in standard English?

Reviewer #1: Yes

Reviewer #2: (No Response)

**********

Reviewer #1: The authors have carefully addressed, revised and responded to all the comments raised by the previous reviewer. The manuscript has been greatly improved, and I recommend its acceptance for publication.

Reviewer #2: (No Response)

**********

what does this mean?). If published, this will include your full peer review and any attached files.

If you choose “no”, your identity will remain anonymous but your review may still be made public.

**Do you want your identity to be public for this peer review?**  For information about this choice, including consent withdrawal, please see our Privacy Policy

Reviewer #1: No

Reviewer #2: No

**********

Attachment Submitted filename: Manuscript.docx

# Editor report: Acceptance letter

**DOI:** 10.1371/journal.pone.0356991.r004

**Authors:** Renwu Wu

PONE-D-26-09521R1

PLOS One

Dear Dr. Lou,

I'm pleased to inform you that your manuscript has been deemed suitable for publication in PLOS One. Congratulations! Your manuscript is now being handed over to our production team.

At this stage, our production department will prepare your paper for publication. This includes ensuring the following:

* All references, tables, and figures are properly cited

* All relevant supporting information is included in the manuscript submission,

* There are no issues that prevent the paper from being properly typeset

You will receive further instructions from the production team, including instructions on how to review your proof when it is ready. Please keep in mind that we are working through a large volume of accepted articles, so please give us a few days to review your paper and let you know the next and final steps.

Lastly, if your institution or institutions have a press office, please let them know about your upcoming paper now to help maximize its impact. If they'll be preparing press materials, please inform our press team within the next 48 hours. Your manuscript will remain under strict press embargo until 2 pm Eastern Time on the date of publication. For more information, please contact onepress@plos.org.

You will receive an invoice from PLOS for your publication fee after your manuscript has reached the completed accept phase. If you receive an email requesting payment before acceptance or for any other service, this may be a phishing scheme. Learn how to identify phishing emails and protect your accounts at https://explore.plos.org/phishing.

If we can help with anything else, please email us at customercare@plos.org.

Thank you for submitting your work to PLOS One and supporting open access.

Kind regards,

PLOS One Editorial Office Staff

on behalf of

Dr. Renwu Wu

Academic Editor

PLOS One
